# Quality-by-design-engineered mitochondrial targeted nanoparticles for glioblastoma therapy†

**DOI:** 10.1039/d4ra04748f

**Published:** 2024-10-28

**Authors:** Akanksha Dahifale, Tejas Girish Agnihotri, Ankit Jain, Aakanchha Jain

**Affiliations:** a Department of Pharmaceutics, National Institute of Pharmaceutical Education and Research (NIPER)-Ahmedabad Palaj Gandhinagar-382355 Gujarat India jainaakanchha83@gmail.com aakanchha.jain@niperahm.res.in; b Department of Pharmacy, Birla Institute of Technology & Science Pilani-333031 Rajasthan India ankit.j@pilani.bits-pilani.ac.in

## Abstract

Glioblastoma (GB, IDH-wildtype) constitutes the most aggressive primary malignant neoplasm with limited treatment modalities due to the blood–brain barrier (BBB) often restricting drug delivery. It also has an overall low survival rate with no curative solution, reinforcing the need for innovative formulation development for effective management of GB. This study explores a novel approach using triphenylphosphonium (TPP^+^)-conjugated chitosan nanoparticles for targeted mitochondrial delivery of temozolomide (TMZ) to GB cells. The conjugated nanoparticles were designed to leverage chitosan's biocompatibility and TPP's mitochondrial targeting ability. TMZ-loaded chitosan nanoparticles were systematically developed and optimized employing a Quality-by-Design (QbD) approach with a screening of factors (Taguchi design) followed by optimization (Box–Behnken design). The optimized nanoparticles had an average particle size of 138.1 ± 5 nm, PDI of 0.242 ± 0.04, and entrapment efficiency of 93.59 ± 3%. Further, a conjugate chitosan-TPP^+^ (CS-TPP^+^) was synthesized and validated, employing varied techniques such as NMR, FTIR, HPLC, zeta potential, and EDAX analysis. *In vitro* drug release in pH 5 phosphate buffer showed a sustained release for nanoparticulate formulations compared to the free drug solution further indicating that conjugation did not alter the release pattern of nanoparticles. With regards to intranasal delivery of the formulation, an *ex vivo* study carried out on goat nasal mucosa demonstrated greater retention of conjugated chitosan nanoparticles on nasal mucosa than free drug solution, and a mucin interaction study also corroborated this finding. *In vitro* cell line studies indicated nanoparticles' cytotoxic potential compared to TMZ solution. Overall, this study highlights the potential of TPP^+^-conjugated chitosan nanoparticles developed strategically for the targeted delivery of TMZ to mitochondria.

## Abstract 2

The current study highlights the potential of systematically developed mitochondrial targeted TPP-conjugated chitosan nanoparticles for delivery of temozolomide in glioblastoma management.

## Graphical abstract

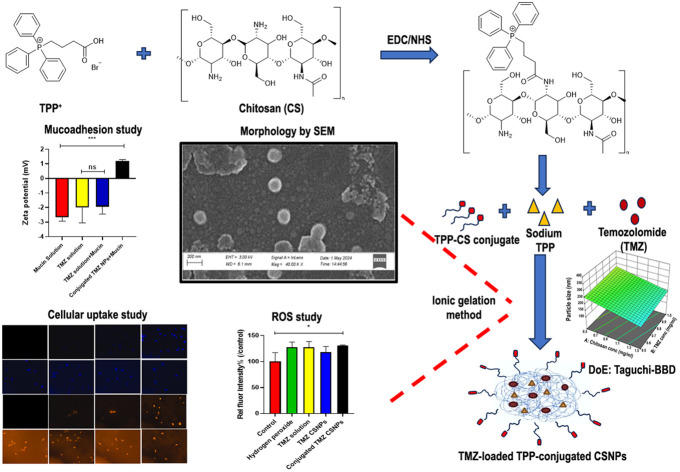

## 1. Introduction

According to WHO classification, 2021, glioblastoma, isocitrate dehydrogenase (IDH)-wildtype (GB), a type 4 astrocytic tumor, affects more than 60% of adults, primarily those in the 55–60 age range.^1–3^ Although it is categorized as a rare tumor, the condition is life-threatening and has a poor diagnosis, with a 14–15 months survival rate after a patient is diagnosed.^4,5^ GB, IDH-wildtype, is considered to be the most common primary malignant neoplasm of the brain, accounting for 54% of all types of gliomas. It is mostly located in the supratentorial region, *i.e.*, frontal, temporal, parietal, and occipital lobes, with higher incidences in the frontal lobe followed by tumors that overlap multiple lobes and then temporal and parietal lobes.^6^ GB, IDH-wildtype is an aggressive and invasive disease; hence, to maximize patient outcomes from treatment, a multi-target approach must be used. Resection of the tumor, radiation therapy, and chemotherapy are now the main treatments for GB, IDH-wildtype.^7^ The standard chemotherapeutic drug authorized for the treatment of GB, IDH-wildtype is temozolomide (TMZ). However, the actual utilization of this compound is limited due to its comparatively short duration in the bloodstream, possible adverse effects on the whole body, and the invasive nature of the related treatment methods.^8^

The presence of the blood–brain barrier (BBB) has been a formidable challenge for neurotherapeutic drug delivery, which makes it difficult for drugs to reach the brain effectively, underpinning the need for innovative ways to get around it and enhance drug delivery. The properties of colloidal-based particle systems referred to as nanocarriers and nanotechnology-based drug delivery systems, such as self-assembly, greater dissolution, delayed drug release, improved drug biological activity, and BBB penetrability, allow them to traverse the BBB.^9,10^ Numerous benefits, including better drug targeting and tumor cell penetrability, which boost therapeutic efficiency, are provided by nanoparticle-based drug delivery systems in the treatment of cancer.^11,12^ One approach involves the surface engineering of nanoparticles through biocompatible coatings. This strategy emphasizes on the optimization of surface ligands and development of stimuli-responsive delivery systems for targeted drug release within the tumor microenvironment.^13,14^ In this regard, targeted drug delivery to mitochondria holds great potential in the clinical intervention of cancer, primarily owing to the crucial involvement of mitochondria in cellular metabolic processes, energy generation, and programmed cell death. The selective delivery of medication to mitochondria can enhance the effectiveness of cancer therapy while minimizing negative effects on non-cancerous cells.^15^ The concept of “mitochondriotropics” often denotes small molecules with a strong innate affinity for mitochondria, as determined by their physicochemical features and reactivity. The most commonly used mitochondrial targeting agents are triphenylphosphonium cation (TPP^+^), dequalinium chloride, peptides, *etc.* One widely employed method for transporting these low-molecular-weight chemicals to mitochondria involves their attachment to mitochondriotropic lipophilic cations, such as TPP^+^. Lipophilic cations possess a delocalized positive charge, which enables them to traverse mitochondrial membranes and concentrate within or close to mitochondria because of the greater mitochondrial membrane potential, which is negative inside. As the driving force behind this mitochondrial-targeted drug delivery seems to be the passive mitochondrial membrane potential difference between normal and cancer cells alone, it is essential to consider associated sensing mechanisms that can detect changes in reactive oxygen species (ROS), pH, glutathione (GSH), the temperature of mitochondria to lessen undesirable effects on other organs. This becomes imperative in cases where drugs are unable to cross cellular membranes, let alone subcellular organelles like mitochondria.^16^ Furthermore, they are crucial to the process of apoptosis mediated by mitochondria.^17,18^

Another approach that circumvents the BBB and helps treat brain diseases is a noninvasive intranasal delivery system.^19^ Intranasal delivery bypasses BBB and provides better brain targeting efficiency, which has been supported by many preclinical findings.^20–23^ Numerous investigators presented preclinical and clinical data suggesting that nose-to-brain delivery is highly effective in treating various brain disorders, including GB, IDH-wildtype treatment approaches. Trigeminal and olfactory nerves are the pathways by which drugs delivered *via* intranasal administration enter the central nervous system.^24^ Drug delivery systems that can stick to the nasal mucosa and pass *via* the olfactory pathway are necessary for nasal delivery of drugs to the brain. A sustained and extended drug administration *via* the nasal route has been described using delivery systems such as mucoadhesive nanoparticles and nanogels which could be effectively targeted to the brain.^25^

In this study, biocompatible chitosan was selected as a mucoadhesive polymer for developing polymeric nanoparticles. The internalization of chitosan (CS) is aided by its positive charges, which are readily bonded with the negatively charged membrane, especially of the mitochondrial membrane. Because of their ability to synthesize adenosine triphosphate (ATP) and transport ions, mitochondria have a significantly greater potential than other organelles. As a result, triphenylphosphine (TPP^+^), a form of lipophilic cationic chemical, may be specifically targeted to mitochondria. A combination of CS, as well as TPP^+^, may smartly target the drug's delivery to the mitochondria, inducing mitochondrial depolarization that would cause a cell stress response, increase in ROS, and ultimately cause cytotoxicity in the tumor cells.^26^

Here, we aim to design triphenylphosphine (TPP^+^)-conjugated chitosan nanoparticles (TPP^+^-CS NPs) to transport TMZ specifically to the mitochondria and increase the antitumor efficacy. To target the mitochondria, grafting triphenyl phosphine onto the surface of (as-prepared) chitosan nanoparticles was done, and TMZ was encapsulated into NPs to study drug loading and the *in vitro* release mechanism. Additionally, *in vitro* cytotoxicity, and intracellular ROS levels were examined to assess the effectiveness of triphenyl phosphonium-linked chitosan nanoparticles for targeted mitochondrial delivery of TMZ.

## 2. Materials and methods

### 2.1 Materials

Temozolomide (TMZ), chitosan low molecular weight (<500 kDa), sodium tripolyphosphate (STPP), (3-carboxypropyl) triphenyl phosphonium bromide (TPP^+^), ROS fluorometric intracellular ROS kit (MAK145) and, dialysis tubing cellulose membrane (MWCO: 12–14 kDa) were obtained from Sigma-Aldrich (Mumbai, India). 1-Ethyl-3-(3-dimethyl aminopropyl) carbodiimide (EDC) and *N*-hydroxy succinimide (NHS) were received from Molychem, India. Minimum Essential Medium (MEM) and penicillin–streptomycin were obtained from Gibco Life Technologies (Grand Island, USA), while trypsin–EDTA solution and Alamar blue assay kit (DAL1100) were procured from Invitrogen (California, USA). All other chemicals employed were of analytical grade. Double distilled water, obtained from the Milli-Q Plus system, was utilized throughout the experiments.

### 2.2 Cell culture

Human glioblastoma cells (U87MG) were obtained from the National Centre for Cell Science (NCCS), Pune, India. The cells were cultured in Minimum Essential Medium (MEM) supplemented with 10% v/v fetal bovine serum (FBS) and penicillin (100 IU mL^−1^)–streptomycin (100 μg mL^−1^) and incubated at 37 °C and 5% CO_2_.

### 2.3 HPLC method development

A standard curve, also referred to as a calibration curve, is created by varying the concentrations of a standard drug, *i.e.*, TMZ, within a linear range. This calibration curve is required to ascertain the analyte concentration in an unknown sample. It demonstrated linearity in two distinct solvents, Milli-Q, and PBS buffer (pH 5), using an established HPLC technique. One mg of TMZ was dissolved in one mL Milli-Q water to prepare the stock solution of 1000 μg mL^−1^. After serial dilutions, a concentration range of 1–250 μg mL^−1^ was obtained from this stock solution. After each concentration's injection, the peak area was calculated to plot the best-fitting line. The regression equation, *y*-intercept, slope, and correlation coefficient were computed. The linearity is considered acceptable if the regression coefficient is higher than 0.999.^27^

### 2.4 Fourier-transform infrared spectral analysis

Using an FTIR-spectrophotometer (Bruker, Germany), the spectra of individual TMZ, chitosan, sodium tripolyphosphate (STPP), and the physical mixture of TMZ, chitosan, and STPP were obtained to evaluate the compatibility between the drug and excipients. The spectra were scanned in the range of wavenumber 4000–400 cm^−1^. Equimolar quantities of TMZ, STPP, and chitosan were combined to form the physical combination, which was further homogenized by trituration with a mortar and pestle. The overlay of spectra then examined the drug's compatibility with the excipients.^28^

### 2.5 Formulation of TMZ-loaded chitosan nanoparticles (TMZ-loaded CSNPs) using QbD approach

Chitosan nanoparticles (CSNPs) were produced by the ionic gelation process, as reported by Pant *et al.*^29^ Sodium tripolyphosphate (STPP) and low molecular weight (LMW) chitosan were used in the synthesis. Briefly, a CS solution in 2% v/v acetic acid was prepared and then agitated for three hours at room temperature (RT) at 1000 rpm. 7 mL of the CS solution was then mixed dropwise with the prepared drug solution and STPP solution in 3 mL of distilled water. The mixture was continuously stirred for one hour using a magnetic stirrer set at 800 rpm. Further, for screening and optimization purpose, the Quality-by-Design approach was utilized.

The Quality by Design (QbD) technique improves the final pharmaceutical product's value and quality. Creating a robust pharmaceutical product with the potential to achieve maximum therapeutic efficacy, quality attributes, and prolonged stability throughout its storage term is the main goal of employing QbD in the pharmaceutical industry. The Quality Target Product Profile (QTPP) and Critical Quality Attribute (CQA) criteria were determined to formulate nanoparticles. A QTPP was developed to direct the development of nanoparticles in line with the QbD approach for drug product development, aiming to improve their biopharmaceutical qualities and offer patients therapeutic advantages. Several patient-centered CQAs were found to meet QTPP requirements. The physical and formulation qualities of nanoparticles as the intended drug product are included in their quality characteristics.^30^ A subset of quality parameters that have a major influence on the product's performance was selected for this particular purpose. Particle size is crucial for the absorption of the nanoparticle. Entrapment efficiency is a measure of the developed delivery system's propensity to encapsulate particles, and zeta potential aids in determining the stability of the formulation. The CQAs of the nanoparticles are directly impacted by the choices made regarding the elements and parameters used as Critical Material Attributes (CMA) and Critical Process Parameters (CPP). The critical material attributes (CMAs), associated with the formulation's constituent parts, and the critical process parameters (CPPs), associated with the manufacturing techniques' instrumental parameters, were defined. This work aimed to optimize TPP^+^-conjugated TMZ-loaded CSNPs for positive surface charge transfer from the nose to the brain using the QbD technique. To create TPP^+^-conjugated TMZ-loaded CSNPs appropriate for nose-to-brain transport, the QTPP and the CQAs were determined before the trials. This formulation included the following characteristics: particle size, polydispersity index (PDI), zeta potential (ZP), and encapsulation efficiency (EE). To optimize the TPP^+^-conjugated TMZ-loaded CSNPs for nose-to-brain delivery through the QbD approach, the QTPP were identified and summarized in ESI Table 1 (Table S1†).

Design expert® software (Stat-Ease, Inc., Version 11, MN, USA) was used to screen and optimize the NPs.

#### 2.5.1. Screening design for preparation of TMZ-loaded CSNPs

Using a 2-level Taguchi orthogonal array design, CMA and CPP that impact product CQA were filtered out. Seven independent variables were chosen based on the preliminary experiment and literature study. CS, STPP, and TMZ concentration were determined as CMA while stirring duration and stirring speed were selected as CPP. Using a two-level strategy, a high (+1) and low (−1) level was chosen for every independent variable to evaluate its impact on CQA. Table 1 displays every independent variable's high and low levels. Design expert® Software (Stat-Ease, Inc., Version11, MN, USA) was used throughout the experimental design. For this experimental design, eight runs were suggested by the software displayed in Table 2. The analysis yielded the following CQAs: particle size (PS), polydispersity index (PDI), and % entrapment efficiency (% EE). For the screening purpose, a half-normal plot and Pareto chart analysis were considered.^31^

**Table 1:** Independent variables and their levels used in Taguchi orthogonal design

Name	Units	Levels	L[1]	L[2]
Chitosan conc.	mg mL^−1^	2	0.5	4
TMZ conc.	mg mL^−1^	2	0.5	2
STPP conc.	mg mL^−1^	2	0.5	2
Stirring speed-1	rpm	2	200	1500
Time-1	h	2	1	4
Stirring speed-2	rpm	2	500	1500
Time-2	min	2	15	60

**Table 2:** Experimental runs of TMZ-loaded CSNPs by employing Taguchi orthogonal design

Std	Factor 1	Factor 2	Factor 3	Factor 4	Factor 5	Factor 6	Factor 7
	A: chitosan conc. (mg mL^−1^)	B: TMZ conc. (mg mL^−1^)	C: STPP conc. (mg mL^−1^)	D: stirring speed-1 (rpm)	E: time-1 (h)	F: stirring speed-2 (rpm)	G: time-2 (min)
1	0.5	0.5	0.5	200	1	500	15
2	0.5	0.5	0.5	1500	4	1500	60
3	0.5	2	2	200	1	1500	60
4	0.5	2	2	1500	4	500	15
5	4	0.5	2	200	4	500	60
6	4	0.5	2	1500	1	1500	15
7	4	2	0.5	200	4	1500	15
8	4	2	0.5	1500	1	500	60

#### 2.5.2. Optimization design for preparation of TMZ-loaded CSNPs

For optimization of TMZ-loaded CSNPs, significant CPP, and CMA were selected according to screening results obtained (Table 3). TMZ-loaded CSNPs were prepared by statistically optimizing the formulation factors using a three-factor, three-level design (3 × 3), *i.e.*, Box–Behnken Design (BBD). Design Expert® software developed and analyzed the experimental design (Stat-Ease, Inc., Version 11, MN, USA). Chitosan concentration (CS conc.: A), temozolomide concentration (TMZ conc.: B), stirring speed after drug and TPP solution addition [stirring speed-2: C], and time to maintain stirring solution after drug and TPP solution addition [time-2: D] were chosen as the independent variables. Three levels of testing were performed on each factor: low (−1), medium (0), and high (+1). Table 4 lists the respective values correlating with these levels. The same CQAs that were selected for Taguchi design, *viz.*, PS, PDI, and % EE, were chosen for BBD. The optimization process for preparing efficient TMZ-loaded CSNPs is guided by this statistical method, which aids in understanding the impact of each element and their interactions on the responses. With BBD, one would find the best circumstances for these formulation variables to get the intended results for the dependent variables.

**Table 3:** Independent variables and their respective levels used in BBD for TMZ-loaded CSNPs

Factors (independent variables)	Levels of variables		
	Low (−1)	Medium (0)	High (+1)
A: CS conc. (mg mL^−1^)	0.5	1	1.5
B: TMZ conc. (mg mL^−1^)	0.5	0.75	1
C: stirring speed-2 (rpm)	800	1000	1200
D: time-2 (min)	20	40	60

**Table 4:** Experimental runs obtained by BBD design during optimization of TMZ-loaded CSNPs

Run	Factor 1	Factor 2	Factor 3	Factor 4
	A: chitosan conc. (mg mL^−1^)	B: TMZ conc. (mg mL^−1^)	C: stirring speed-2 (rpm)	D: time-2 (min)
1	1	0.5	1000	20
2	1.5	0.75	1200	40
3	1	0.75	1000	40
4	1	0.75	1200	20
5	1	0.75	1000	40
6	1.5	0.75	1000	20
7	1.5	0.75	1000	60
8	0.5	0.75	1000	20
9	1	1	1000	60
10	1.5	1	1000	40
11	1	0.75	1200	60
12	0.5	1	1000	40
13	0.5	0.5	1000	40
14	0.5	0.75	1000	60
15	1	0.5	1000	60
16	1	1	1200	40
17	1.5	1	1000	40
18	1	0.75	800	60
19	1	0.75	1000	40
20	1	1	800	60
21	1	0.5	1200	40
22	1	0.5	800	40
23	1.5	0.5	1000	40
24	1.5	0.75	800	40
25	0.5	0.75	1000	40
26	0.5	0.75	800	40
27	1	0.75	800	20

The two extreme levels of CS and TMZ conc. were chosen on performing preliminary and screening testing of the impact of factors above on PS, PDI, and % EE. Based on the preliminary study and screening study, broader ranges were selected. Software generated the medium levels of the enlisted factors based on the chosen levels. Using BBD, with 3 repetitions of the center point, 27 runs were obtained (Table 4) performed, and term significance was estimated by analysis of variance (ANOVA). Probability *p*-values (*p* < 0.05) denoted significance.^32^

#### 2.5.3. Model validation

Independent variables were optimized by graphical methods, wherein the optimized formula was designated from the design space in the overlay plot. The criteria for selecting the optimized region were to attain a smaller particle size, narrow particle distribution (PDI), and larger % EE.

### 2.6 Synthesis of triphenylphosphonium-chitosan (TPP^+^-CS) conjugate

NPs were conjugated by the method adopted by Huang *et al.* with minor modifications.^33^ A 100 mg quantity of low molecular weight-chitosan (LMWT-CS) was dissolved in 100 mL of acidified water (2% acetic acid solution; pH 2.5) under stirring until complete homogenization (3 h). Simultaneously, triphenylphosphonium (TPP^+^) was activated for 2 h using an aqueous solution of EDC/NHS. The activated TPP^+^ solution was then dropped into the CS solution and stirred overnight at low temperatures (CS : TPP^+^ in ratio 2 : 1). Subsequently, the solution pH was adjusted to 9 using 1 N NaOH inducing precipitation of conjugated CS polymer. The precipitate was collected *via* centrifugation (10 000 rpm, 20 min). The pellet subsequently was kept for dialysis (MWCO: 12–14 kDa) for 2 days to remove unconjugated materials. Then, the collected pellet was resuspended in water, frozen, and lyophilized.

### 2.7 Structural validation of TPP^+^-CS conjugate

#### 2.7.1. Proton nuclear magnetic resonance (^1^H NMR)

The sample was prepared by dissolving TPP^+^-CS conjugate (5 mg) in CH_3_COOD and D_2_O solution. ^1^H NMR spectrum was recorded at ambient temperature on an Avance 500 MHz NMR spectrometer (Bruker Avance 500 Coventry, UK).

#### 2.7.2. Fourier-transform infrared spectroscopy analysis

The functional groups' elucidation of TPP^+^-CS conjugate was determined by FTIR spectroscopy with scanning from 4000 to 400 cm^−1^ (Frontier IR, PerkinElmer, USA).^34^

#### 2.7.3. Zeta potential study

Chitosan and TPP^+^-CS were dissolved in an aqueous acetic acid solution using agitation to form a solution (1 mg mL^−1^). The zeta potential was measured by Zetasizer Nano ZS (Malvern, United Kingdom) at 25 °C.^35^

#### 2.7.4. % conjugation efficiency determination

To determine the % conjugation efficiency, a reaction mixture containing TPP^+^-CS conjugate was centrifuged for 10 min (10 000 rpm, 4 °C). Then, the supernatant was separated and injected into HPLC (HPLC 1260 Infinity, Agilent Technologies, CA, USA). Unconjugated TPP^+^ concentration was calculated by plotting the linearity of TPP^+^ with the developed HPLC method. This was further used to determine % conjugation efficiency.^36^

HPLC method was developed using HPLC (1260 Infinity, Agilent Technologies, CA, USA). Eclipse Plus C18 column (4.6 mm × 250 mm, 5 μm) was utilized for separation using a mobile phase consisting of ammonium formate buffer (10 mM) as an aqueous phase and acetonitrile (ACN) as an organic phase, respectively. Keeping the injection volume at 10 μL, analysis was done at 267 nm.

#### 2.7.5. Elemental analysis by SEM/EDAX

In the preparation for SEM/EDX analysis, the sample of TPP^+^-CS was initially subjected to lyophilization to convert it into a dry powder form. Subsequently, the lyophilized powder was coated with a thin layer of gold. This gold coating was applied to enhance the sample's electrical conductivity and improve the quality of the SEM imaging. The SEM/EDX analyses were then conducted on the gold-coated powder to investigate its morphological and elemental characteristics.

### 2.8 Preparation of TPP^+^-conjugated TMZ-loaded CSNPs

The conjugated TMZ-loaded CSNPs were made using a similar process to the one previously optimized for producing chitosan nanoparticles. The lyophilized conjugated CS-TPP^+^ polymer (1 mg mL^−1^) was dissolved in an aqueous solution of acetic acid using agitation [1000 rpm (stirring speed-1), 3 h (time-1)]. TPP (1.5 mg mL^−1^) and TMZ solutions (0.5 mg mL^−1^) were also prepared in Milli-Q water. Subsequently, the TMZ and TPP solutions were added dropwise to the CS-TPP^+^ solution while it was continuously stirred at 800 rpm (stirring speed-2) for 1 h (time-2), resulting in the synthesis of conjugated nanoparticles.

### 2.9 Characterization of TMZ-loaded CSNPs and TPP^+^-conjugated TMZ-loaded CSNPs

#### 2.9.1. Particle size, PDI, and zeta potential determination

Particle size and the polydispersity index (PDI) of both TMZ-loaded CSNPs and TPP^+^ conjugated TMZ-loaded CSNPs were evaluated using a zeta sizer (Nano ZS, Malvern, United Kingdom). Dynamic light scattering, which measured the distribution of light intensity change caused by nanoparticles repeatedly exposed to Brownian motion, served as a base for this concept. The Laser Doppler Electrophoresis technique was utilized to assess the electrophoretic mobility of nanoparticles and calculate the zeta potential of colloids dispersed in a liquid. For evaluation, nanoparticulate formulation was used without any dilution. The light scattering was measured at ninety degrees, and the system was kept at room temperature. Every measurement was carried out three times.^37^

#### 2.9.2. % EE determination of TMZ-loaded CSNPs and TPP^+^-conjugated TMZ-loaded CSNPs

% EE was determined by using an indirect method. The % EE of both formulations, *viz.*, TMZ-loaded CSNPs and TPP^+^-conjugated TMZ-loaded CSNPs, was determined from supernatant collected upon centrifugation at 12 000 rpm for 30 min at RT.^32,38^ The quantity of free TMZ in the clear supernatant was estimated by the HPLC method at 330 nm. Free TMZ conc. was calculated by a previously developed TMZ calibration curve. Then, the %EE was calculated according to the following equation,1
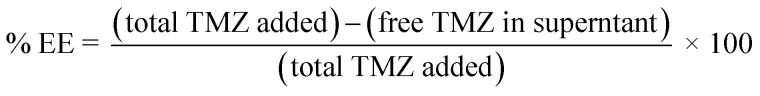


#### 2.9.3. % drug loading determination of TMZ-loaded CSNPs and TPP^+^-conjugated TMZ-loaded CSNPs

The lyophilization technique determined the % DL of TMZ-loaded CSNPs and TPP^+^-conjugated TMZ-loaded CSNPs. The prepared nanoparticles suspension was kept for freeze drying for 12 h. After that, the freeze-dried sample was kept in a lyophilizer to get a lyophilized form of nanosuspension. The total weight of the lyophilized form of nanoparticles was then taken, and % DL was computed by using the following formula:^39^2
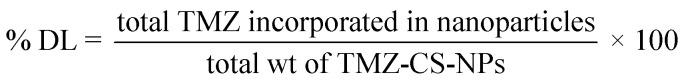


#### 2.9.4. *In vitro* drug release study

The *in vitro* drug release of the TMZ drug solution, TMZ-loaded CSNPs, and TPP^+^-conjugated TMZ-loaded CSNPs was done by the dialysis bag method. 1 mg TMZ equivalent of optimized TMZ-loaded CSNPs and TPP^+^-conjugated TMZ-loaded CSNPs was suspended in 1 mL MilliQ-water and taken in the dialysis bag, which was tied at both ends and immersed in a beaker containing 25 mL of PBS (pH 5; to mimic tumor physiological environment) as a release medium. For the TMZ drug solution, 1 mg was dissolved in 1 mL of Milli-Q water and taken in the dialysis bag. It was then put on a magnetic stirrer at 200 rpm and the temperature was maintained at 37 ± 1 °C throughout the experiment. At definite time intervals, (0.25, 0.5, 0.75, 1, 2, 4, 8, 12, 24 h) sample was withdrawn, and the release medium was replaced with equal amounts of fresh PBS (pH 5). The TMZ amount released was analyzed at 330 nm using an HPLC. The study was done in triplicate with the mean ± SD.^40^

#### 2.9.5. Drug release kinetics

The *in vitro* release data for TMZ-loaded CSNPs and TPP^+^-conjugated TMZ-loaded CSNPs were fitted in different mathematical models like zero order, first order, Higuchi, Korsmeyer–Peppas, Hixson–Crowell, Weibull, *etc.*, to investigate the drug release kinetics utilizing the DD Solver software.^41^

### 2.10 Morphological assessment of TMZ-loaded CSNPs and TPP^+^-conjugated TMZ-loaded CSNPs

The morphology of nanoparticle suspension was assessed using FE-SEM (SIGMA S300, Zeiss). During sample preparation, nanoparticles need to be diluted to get an image of discrete particles with better resolution and clarity. Nanoparticle formulation was diluted using Milli-Q water, and one drop of nanoparticles diluted solution was taken on a glass slide, air dried for 10 min, and again kept for drying overnight using the vacuum dryer. Then, samples were coated with conductive material (gold-coated). Because gold coating increases sample stability, protects samples, improves resolution and image quality, and enhances conductivity, it is essential to have accurate and dependable imaging of various materials in FE-SEM analysis. Images were captured under the accelerated electron voltage of 40 kV.^42^

### 2.11 Stability study

The TMZ-loaded CSNPs and TPP^+^-conjugated TMZ-loaded CSNPs were stored at 25 ± 1 °C and 4 ± 1 °C for 28 days. The aliquots of NPs were collected at different time intervals (0, 1, 3, 7, 14, and 28 days). The samples were subsequently characterized for particle size, PDI, and zeta potential. The results were reported as mean ± S. D. (*n* = 3).^43^

### 2.12 *Ex vivo* drug permeation study

Franz diffusion cells with a contact surface area of 2.26 cm^2^ and a receiver compartment capacity of 15 mL were used to conduct an *ex vivo* permeation study on goat nasal mucosa. It has been used for studies because goat nasal mucosa has similar physiological characteristics to human nasal mucosa. Fresh goat nasal mucosa was procured from a slaughterhouse. Adherent mucus and adipose tissues were meticulously dissected, and the tissue was trimmed to appropriate dimensions for subsequent mounting. The mounting orientation ensured the mucosal surface faced the donor compartment, while the serosal surface opposed the receiver compartment. To establish electrophysiological equilibrium, the tissue was pre-incubated for 15 min at 37 °C with saline solution filling both compartments. Subsequently, 1 mL of TMZ solution was introduced into the donor compartment. At predetermined intervals (5, 10, 15, 30, 60, 120, 240, 360, and 480 min), 1 mL aliquot was collected from the receiver compartment and immediately replaced with an equal volume of fresh media. The same procedure was performed for TPP^+^-conjugated TMZ-loaded CSNPs. HPLC method was developed to assess unknown sample concentration for drug penetration. The isocratic method was used, and the mobile phase composition was selected as a 90 : 10 ratio of phosphate buffer (pH 6.5) and acetonitrile at 330 nm and 1.0 mL min^−1^ flow rate with a run time of 7 min.

### 2.13 Mucoadhesion study

A mucin binding assay was employed to assess the mucoadhesive potential of formulated nanoparticles based on alteration in their zeta potential. Deionized water was used to dissolve 1% w/v porcine mucin powder. The mucin solution was centrifuged at 5000 rpm for 10 min, and the resulting supernatant was utilized for mucoadhesion studies. An equal amount of formulation (TMZ solution and conjugated formulation) and 1% w/v mucin supernatant were mixed with a vortexer for one minute. The mixture was then left for four hours. Then, the zeta potential of the mucin solution was noted. Zeta potential measurement was performed on each combination with Zeta Sizer (Nano ZS, Malvern, United Kingdom). Mucoadhesion was evidenced by the change in zeta potential detected.^44^ The experiment was done in triplicate.

### 2.14 Cytotoxicity study

Alamar blue assay (DAL1100, Invitrogen, USA) was performed to assess the cytotoxicity of the prepared formulation. U87MG cell line was used and cultured in Minimum Essential Medium (MEM) with 10% fetal bovine serum albumin (10% FBS). They were maintained in an incubator at 37 °C and 5% CO_2_. Cells were seeded in 96 well plates at a density of 10 000 cells per well. Then cells were treated with different concentrations of TMZ solution ranging from 1 to 5000 ppm for 24 h. The cells were also treated with TMZ-loaded CSNPs (concentration range: 0.1–100 ppm) and TPP^+^-conjugated TMZ-loaded CSNPs (concentration range: 1–100 ppm) diluted in a serum-free MEM medium. Control cells were treated with serum-free media without any treatment for 24 h. After 24 h of treatment, 10 μL of Alamar Blue Reagent (10 μg mL^−1^) was added to each well and kept in an incubator for 4 h. The fluorescence intensity of the solutions in each well was measured using a multimode UV microplate reader (Varioskan LUX, Thermo Fischer Scientific) at Ex/Em 560–590 nm. The cell viability was calculated by using formula,^45^3
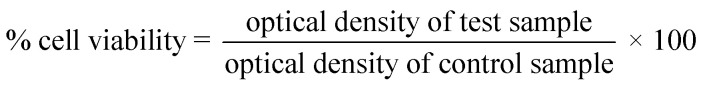


### 2.15 Intracellular reactive oxygen species (ROS) study

Intracellular ROS level was assessed by a fluorometric intracellular ROS kit (Sigma-Aldrich, MAK145). U87MG cell lines were seeded (MEM with 10% FBS) in a 96-well plate at a density of 1 × 10^4^ cells per well. The cells were incubated at 37 °C and 5% CO_2_ overnight and then treated with master-mix solution (provided with the kit) for 1 h. The treatment (TMZ solution, TMZ-loaded CSNPs, and TPP^+^-conjugated TMZ-loaded CSNPs at their IC_50_ value) was then given for a specified period *i.e.*, 3 h, 6 h, and 12 h. The next proceeding was done according to the manufacturer's protocol and the fluorescence intensity was measured with a multimode plate reader (Varioskan LUX Multimode, Thermo Fisher Scientific, USA) at excitation/emission wavelength at 520/605 nm. U87MG cells with media were considered as a control while hydrogen peroxide solution was taken as a positive control. The experiment was done in triplicate.

### 2.16 Cellular uptake study

Cellular uptake study was done to track whether the prepared nanoparticles penetrate into the cells or not. To track the fate of TMZ, rhodamine B, a reporter dye (0.5% w/v) was integrated into nanoparticles (unconjugated as well as conjugated). It was then subjected to centrifugation to separate TMZ-loaded CSNPs from unloaded rhodamine B. Briefly, U87MG (50 000 cells per well) were seeded in 6-well plates that were covered with 18 mm coverslips. The were incubated overnight at 5% CO_2_ at 37 °C and then treated with TMZ solution, unconjugated TMZ-loaded CSNPs, and TPP^+^-conjugated TMZ-loaded CSNPs for 2 h and 4 h. Cells were fixed with 4% paraformaldehyde solution in PBS for 15 min and then stained with 10 μg of DAPI in PBS for 10 min followed by 2 times washing with PBS. Cellular uptake of nanoparticles was then observed by fluorescent microscopy.^46^

### 2.17 Statistical analysis

All studies were performed in triplicate (mean ± S. D.) and graphs were made using Graph Pad Prism (Version 8.0.2). One-way Analysis of Variance (ANOVA), a parametric test was utilized for multigroup analysis with the Tukey–Kramer multiple comparison posthoc test (*p < 0.05, **p < 0.01, ***p < 0.001 represented statistically significant difference).

## 3. Results and discussion

### 3.1 Spectrophotometric determination of TMZ by HPLC

HPLC method was employed to analyze the unknown concentration of TMZ in solution and formulation. The maximum absorption wavelength (*λ*_max_) of TMZ was found at 330 nm and the retention time was observed at 4.3 min. High correlation coefficients (*R*^2^ values of 0.9993 and 0.9998) indicate the linearity of the calibration curves for TMZ in both Milli-Q water and PBS buffer (pH 5), respectively. The HPLC chromatogram of TMZ is shown in ESI Fig. S1(a),† and the linear range of TMZ solution in Milli-Q water (1–250 μg mL^−1^) is shown in Fig. S1(b),† with regression equation as *y* = 27.227*x* + 20.119, with an *R*^2^ value of 0.9993. Fig. S1(c)† shows the linearity range of TMZ solution (1–100 μg mL^−1^) in PBS buffer (pH 5). The regression equation is *y* = 23.448*x* + 12.047, and its *R*^2^ value was found to be 0.9998.

### 3.2 Fourier-transform infrared spectral analysis

**Fig. 1:**
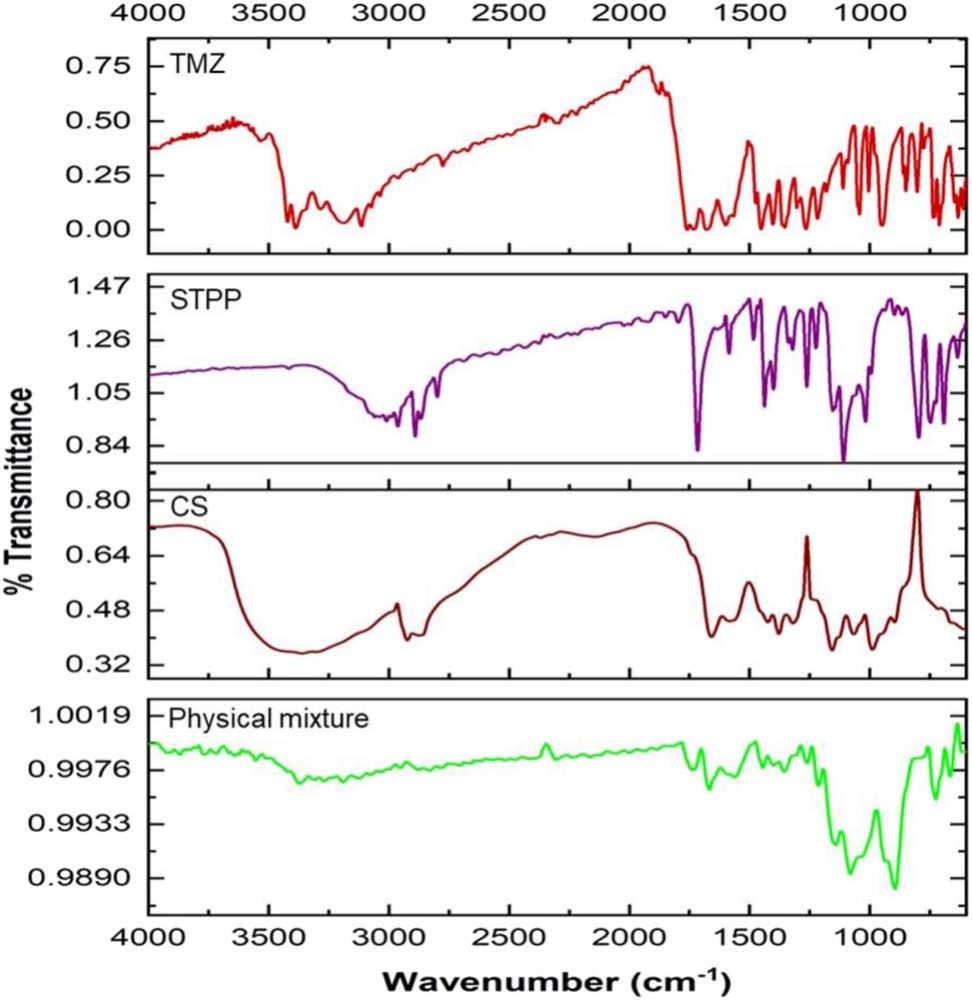
(a) Overlay of FTIR spectra of TMZ, STPP, CS, and their physical mixture as a pre-formulation study indicating compatibility between TMZ and excipients.

Fourier transform infrared (FTIR) spectroscopy is a powerful analytical tool for characterizing drug molecules and excipients. By identifying their unique vibrational fingerprint regions in the infrared spectrum, FTIR spectroscopy allows for detecting potential interactions between these components. The spectra of drug and excipients, *viz*; CS, STPP, and TMZ, were obtained individually, and similarly, the spectrum of the physical mixture of drug and excipients was also taken. The overlay of spectra is represented in Fig. 1. The characteristic peaks of TMZ in the region of 3384.36 cm^−1^ (–NH stretching), 3038.32 cm^−1^ (–CH2), 1732.05 cm^−1^(–NH–C=O), 1678.72 cm^−1^ (amide bending), and 1451.67 cm^−1^ (–CH bending) were found to be retained in the spectrum of physical mixture with no addition or deletion of peaks indicating that there was no chemical interaction between drug and excipients thus, signifying the compatibility of TMZ with CS and STPP. A similar observation was seen for CS (characteristic peaks of 3358, 2924, 1655, 1575, 1378, 1156, 898 cm^−1^) and STPP (characteristic peaks of 2890, 1715, 1261, 1153, 1108 cm^−1^) where all significant peaks were retained in the spectrum of the physical mixture, further confirming the compatibility.

### 3.3 Formulation of TMZ-loaded CSNPs

CSNPs were formulated using biocompatible and biodegradable polymer like CS and STPP as crosslinking agent. By creating crosslinks with the CS polymer, the STPP helped to entrap TMZ in the polymer matrix and promote the formulation of CSNPs. The resultant mixture was continuously stirred for 1 h using the ionic gelation process to formulate TMZ-loaded CSNPs.

#### 3.3.1. Screening of factors for preparation of TMZ-loaded CSNPs

Using a Taguchi orthogonal array design, the factors that affected the response variables to a greater extent were selected.

##### 3.3.1.1 Particle size

**Fig. 2:**
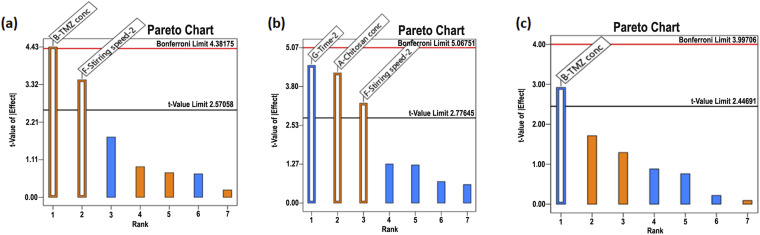
Pareto chart analysis during screening by Taguchi design (a) particle size, (b) PDI, (c) % entrapment efficiency.

The particle size of TMZ-loaded CSNPs was distributed in a range of 318–3515 nm. The particle size of NPs suspensions was analyzed using Zetasizer (Nano ZS, Malvern, United Kingdom). Table 5 shows the particle size of the TMZ-loaded CSNPs for the formulations batch 1 to 8. Batch 1–3 and 5–8 had larger particle sizes than batch 4. From these results, the level of factors has been selected for further optimization purposes. Out of all selected factors, TMZ conc. (*F* value of 19.60) and stirring speed-2 (*F* value of 11.96) showed a positive effect on particle size demonstrated by the Pareto chart and ANOVA Table of Taguchi design. Both the factors crossed the *t*-value limit in the Pareto chart, signifying the possible impact on particle size. Further *p* values of both factors were less than 0.05, again highlighting their significant impact on particle size. (Fig. 2(a) and ESI Table S2(a)†).

**Table 5:** Taguchi design table with response variables during screening of TMZ-loaded CSNPs

Run	Factor 1	Factor 2	Factor 3	Factor 4	Factor 5	Factor 6	Factor 7	Response 1	Response 2	Response 3
	A: chitosan conc. (mg mL^−1^)	B: TMZ conc. (mg mL^−1^)	C: STPP conc. (mg mL^−1^)	D: stirring speed-1 (rpm)	E: time-1 (h)	F: stirring speed 2 (rpm)	G: time-2 (min)	Particle size (nm)	PDI	Entrapment efficiency (%)
1	4	0.5	2	1500	1	1500	15	748.7	0.267	59.2787
2	4	2	0.5	1500	1	500	60	1594	0.683	14.1182
3	4	2	0.5	200	4	1500	15	3515	0.293	28.0109
4	0.5	0.5	0.5	200	1	500	15	318.9	0.489	56.5438
5	0.5	2	2	200	1	1500	60	3132	0.857	38.3676
6	0.5	2	2	1500	4	500	15	1336	1	46.4461
7	4	0.5	2	200	4	500	60	726.8	0.692	98.3426
8	0.5	0.5	0.5	1500	4	1500	60	1493	0.832	63.5691

##### 3.3.1.2 PDI

The PDI of TMZ-loaded CSNPs was distributed in a wide range from 0.26 to 0.85. The PDI of NPs was analyzed using Zetasizer. Table 5 shows the PDI of the CSNPs for the formulation batch 1–8. The PDI obtained in batches 2 and 5–8 was larger than those obtained in batches 1 and 3–4. The Pareto chart and ANOVA table (Fig. 2(b) and Table S2(b)†) indicated that the time-2 (*F* value of 20.15), chitosan conc. (*F* value of 17.99), and stirring speed-2 (*F* value of 10.60) showed a significant impact on PDI. Time-2 showed a negative impact, meaning as time increased, there was a decrease in the PDI, but chitosan conc. and stirring speed-2 showed positive effects, meaning as CS conc. or stirring speed-2 increased, there was an increase in PDI.

##### 3.3.1.3 % entrapment efficiency (% EE)

The % EE of TMZ-loaded CSNPs was obtained in a wide range from 14.11% to 98.34%. The entrapment of TMZ in CSNPs suspensions was analyzed using the HPLC method. Table 5 shows the entrapment efficiency of the TMZ-loaded CSNPs for the formulation batches 1–8. The entrapment obtained in batches 1, 4, 7, and 8 was larger than those obtained in batches 2, 3, 5, and 6. By analyzing the Pareto chart and ANOVA Table (Fig. 2(c) and Table S2(c)†), it was found that the TMZ conc. (*F* value 8.52) showed a significant impact on entrapment efficiency. The % EE decreases with increasing TMZ concentration. This negative relationship between TMZ conc. and % EE suggests that % EE falls with increasing TMZ concentration.

#### 3.3.2. Optimization of TMZ-loaded CSNPs by Box–Behnken design (BBD)

The particle size, PDI, and % EE were modeled using linear regression analysis. Three models were considered, *viz.*, linear, two-factor interaction (2FI) and quadratic. The model with the highest adjusted *R*^2^ and prediction *R*^2^ was selected for each response. ANOVA was then performed to identify statistically significant terms (*p* < 0.05) within the chosen model. Nonsignificant terms were removed through model reduction to enhance model accuracy and improve prediction *R*^2^.

Design Expert® software (version 13) was employed to evaluate model fit based on adjusted *R*^2^ and prediction *R*^2^. For PS, a linear model provided the optimal fir. On the other hand, quadratic models were chosen for both PDI and % EE. These selections were made based on the models with the highest adjusted and predicted *R*^2^ values. After model reduction, the final equations for the responses related to different factors and interactions in terms of coded variables were as follows,

4 PS = + 215.80 − 40.73 (CS conc.) + 12.62 (TMZ conc.) − 7.26 (stirring speed-2) − 56.98 (time-2)

5 PDI = +5059 + 0.0400 (CS conc.) + 0.0121 (TMZ conc.) + 0.0610 (stirring speed-2) − 0.009^2^ (time-2) − 0.1197 (CS conc. × TMZ conc.) + 0.0462 (CS conc. × stirring speed-2) − 0.0345 (stirring speed-2)^2^ − 0.0595 (Time-2)^2^

6 % EE = +76.93 + 1.62 (CS conc.) − 0.3585 (TMZ conc.) + 3.56 (time-2) − 11.36 (TMZ conc. × time-2) − 11.99 (CS conc.)^2^

##### 3.3.2.1 Influence of investigated factors on particle size

**Fig. 3:**
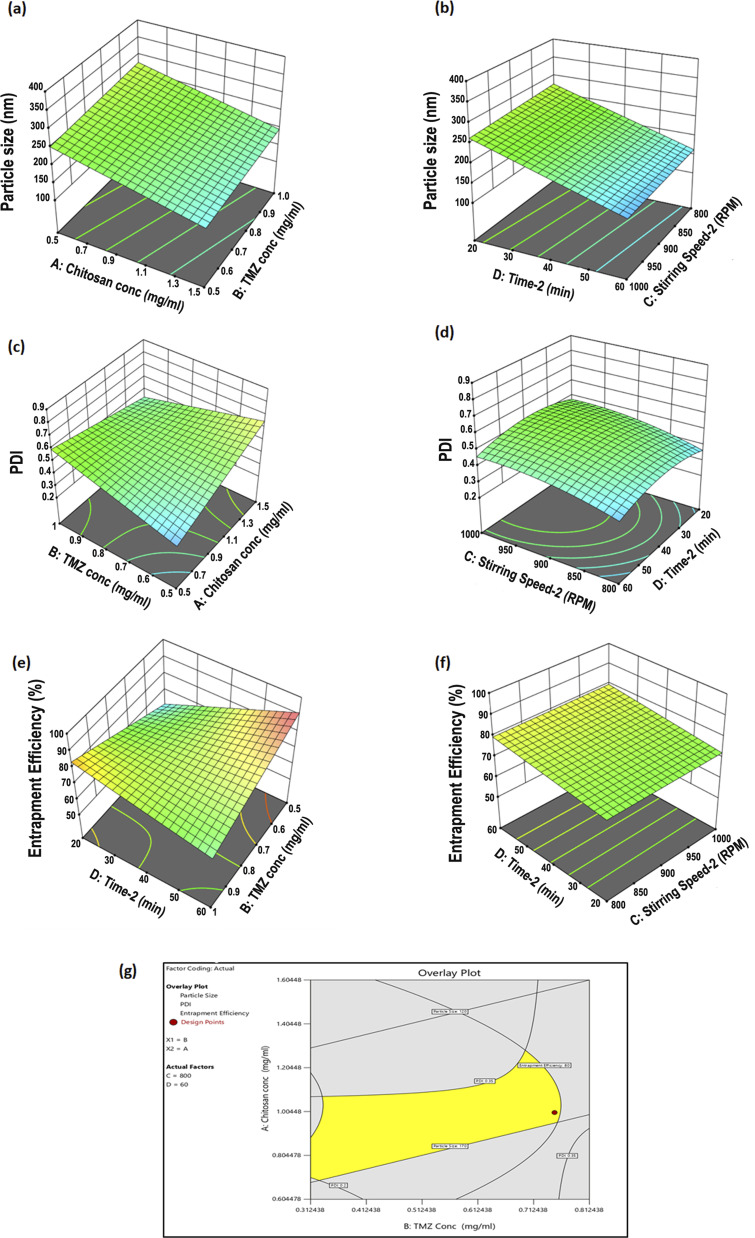
The 3D surface plot for main effects and interactions among CS conc., TMZ conc., stirring speed-2, and time-2 on (a and b) particle size with inverse correlation with CS concentration and time-2, (c and d) PDI with positive correlation with TMZ concentration and CS concentration, (e and f) % entrapment efficiency with positive correlation with TMZ concentration, (g) overlay plot showing design space as model validation.

ANOVA analysis showed that CS conc. (A), and time-2 (D) were the significant factors affecting average PS (*p* < 0.05), while TMZ conc. (B) and stirring speed (C) had no significant effect on particle size (Table S3(a)†). As seen in the 3D surface plot illustrated in Fig. 3(a and b), both factors (CS and time-2) were inversely correlated with particle size. The results demonstrated that particle size was significantly affected by CS conc. and time-2. The optimum formula with the smallest particle size was obtained when the CS conc. was 1 mg mL^−1^, and the particle size was found to increase below and above this conc. Below this concentration, the concentration of CS was not sufficient to form effective cross-linkage with STPP. With a CS–STPP ratio of 1 : 1.5, the particle size decreased due to the enhanced density of cross-linkage between CS and STPP. However, increasing the TMZ conc. above a certain limit could also lead to particle size enlargement due to the accumulation of excess TMZ on the surface of the NPs. Also, time-2 helps in reducing the size of the particles, but if it falls below a specific point, aggregation forms and the size of the particles grows. Optimum size nanoparticles (<200 nm) were obtained at 60 min stirring.

##### 3.3.2.2 Influence of investigated factors on PDI

The PDI of TMZ-loaded CSNPs varied from 0.25 to 0.69, as shown in Table 6. ANOVA analysis indicated that all factors, A, B, C, and D, significantly affected the PDI of the prepared NPs (*p* < 0.05) respectively (Table S3(b)†). The main effects of A, B, and C on PDI are represented by the 3D surface plots in Fig. 3(c and d). PDI significantly increased by increasing the CS concentration and stirring speed-2. A decrease in the TMZ concentration and an increase in stirring time-2 were associated with a significant decrease in PDI.

**Table 6:** Formulation batches with response variables employing BBD design for optimizing TMZ-loaded CSNPs

Runs	Factor 1	Factor 2	Factor 3	Factor 4	Response 1	Response 2	Response 3
	A: chitosan conc. (mg mL^−1^)	B: TMZ conc. (mg mL^−1^)	C: stirring speed-2 (rpm)	D: time-2 (min)	Particle size (nm)	PDI	EE (%)
1	1	0.5	1000	20	349.23	0.49	57.12
2	1.5	0.75	1200	40	150.6	0.6	78.91
3	1	0.75	1000	40	106.9	0.43	75.54
4	1	0.75	1200	20	303.1	0.38	83.94
5	1	0.75	1000	40	370	0.69	69.54
6	1.5	0.75	1000	20	169.4	0.48	54.62
7	1.5	0.75	1000	60	205.2	0.49	74.08
8	0.5	0.75	1000	20	212.8	0.39	57.48
9	1	1	1000	60	109.3	0.52	66.64
10	1.5	1	1000	40	135.3	0.56	64.01
11	1	0.75	1200	60	102.6	0.25	81
12	0.5	1	1000	40	329.3	0.56	63.22
13	0.5	0.5	1000	40	209.4	0.36	64.41
14	0.5	0.75	1000	60	188.2	0.4	67.36
15	1	0.5	1000	60	114.2	0.43	89.55
16	1	1	1200	40	239.9	0.38	59.79
17	1.5	1	1000	40	214.8	0.47	69.1
18	1	0.75	800	60	117.5	0.38	81.29
19	1	0.75	1000	40	104.5	0.4	78.19
20	1	1	800	60	201.9	0.36	73.49
21	1	0.5	1200	40	138.3	0.33	83.61
22	1	0.5	800	40	274.8	0.35	77.65
23	1.5	0.5	1000	40	147.8	0.8	54.17
24	1.5	0.75	800	40	178.9	0.41	70.68
25	0.5	0.75	1000	40	303.1	0.43	67.83
26	0.5	0.75	800	40	282.1	0.38	59.65
27	1	0.75	800	20	231.3	0.36	84.4

##### 3.3.2.3 Influence of investigated factors on % EE

The % EE of TMZ-loaded CS NPs ranged from 54.62% to 89.55% as shown in Table 6, indicating that the NPs highly entrapped TMZ. As shown by the 3D surface plot in Fig. 3(e and f), TMZ conc. and chitosan conc. significantly affected % EE. The results of the ANOVA showed that the TMZ conc. and time-2 had a significant impact on % EE (*p* < 0.05). Additionally, the chitosan conc. demonstrated a significant effect on % EE (*p* < 0.05) (Table S3(c)†). Increasing the CS conc. led to a decreased % EE. This could be due to the increased viscosity of CS solution with higher CS conc., which restricted loading the TMZ cargo into the CS matrix.

#### 3.3.3. Model validation

The selection of the optimal formulation aims to achieve minimal particle size, narrow polydispersity index (PDI), and high encapsulation efficiency. Fig. 3(g) depicts an overlay plot. The yellow region represents the optimal design space that satisfies the desired formulation characteristics. Conversely, the grey region encompasses areas where the predicted responses fall outside these criteria. The chosen optimized formula comprised four independent variables *viz.*, *X*_1_ (CS concentration), *X*_2_ (TMZ concentration), *X*_3_ (stirring speed-2), and *X*_4_ (time-2). This formulation yielded predicted values for three dependent variables (size: 148.77 nm, PDI: 0.347, and % EE: 90.28) (Table 7).

**Table 7:** Validation model summary statistics of quadratic model

Responses	*R* ^2^	Adjusted *R*^2^	Predicted *R*^2^	Constrains	Predicted results	Observed results
Particle size (nm)	0.3538	0.2364	0.1222	Minimize	148.77	143.96
PDI	0.7384	0.6222	0.4970	Minimize	0.347	0.34
% EE	0.5639	0.4601	0.2665	Maximize	90.28	93.29

### 3.4 Conjugation of TPP^+^-CS conjugate

**Scheme 1:**
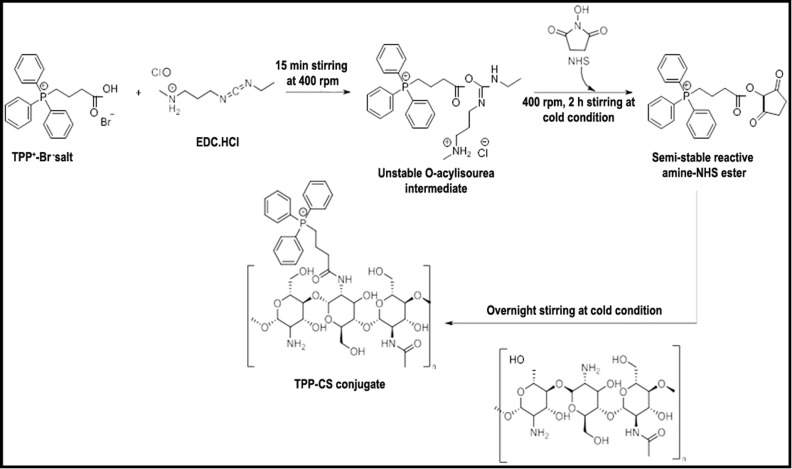
Reaction scheme of CS-TPP^+^-conjugate synthesis wherein EDC·HCl chemically reacts with TPP^+^ Br^−^ salt to form unstable *O*-acyl-isourea intermediate on 15 min magnetic stirring at 400 rpm followed by addition of NHS under stirring at 400 rpm for 2 h under cold conditions to form amine NHS-ester. Finally, TPP-CS conjugate gets formed after addition of NHS ester into acidified aqueous chitosan solution with overnight stirring.

The protocol described in Scheme 1 of the chemical synthesis technique was followed in the synthesis of the TPP^+^-CS conjugate. TPP^+^ carboxylic group activation was first accomplished by interaction with coupling chemicals, specifically EDC·HCl and NHS. Following a series of steps, an amine-reactive *O*-acyl-isourea intermediate was formed, which later transformed into the more stable amine-reactive NHS ester. After activation, the required conjugate (TPP^+^-CS) was formed and linked by a stable amide bond when the NHS ester was added to an acidified aqueous solution of CS-polymer. The grafted polymer was isolated by centrifugation and subsequently subjected to lyophilization, resulting in a brownish-yellow powder.

### 3.5 Structural validation of TPP^+^-CS conjugate

**Fig. 4:**
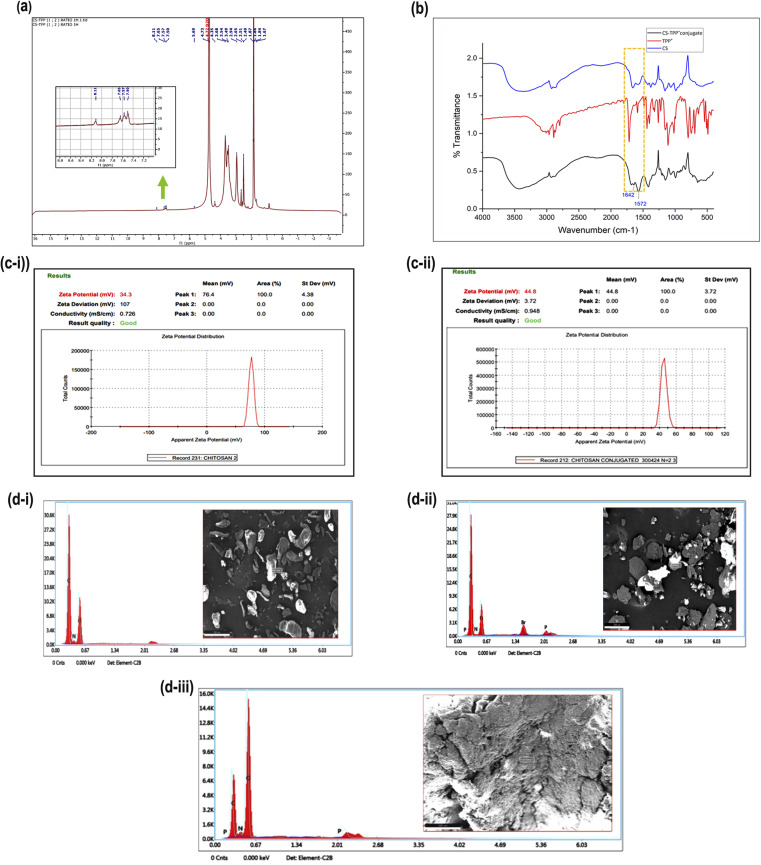
Structural validation of CS-TPP^+^-conjugate by different analytical techniques (a) ^1^H NMR spectrum of CS-TPP^+^ conjugate, (b) overlay of FTIR spectra of CS, TPP and CS-TPP^+^ conjugate, (c) zeta potential measurement of (i) CS solution (ii) conjugated CS-TPP^+^ solution, (d) elemental analysis by SEM/EDAX (i) CS (ii) TPP (iii) CS-TPP^+^ conjugate.

Fig. 4(a) displays the ^1^H NMR spectrum of TPP^+^-CS. The existence of signals at the aromatic area of the spectrum between 7.80 and 8.57 ppm, which are relevant to the three phenyl rings of the added triphenylphosphonium groups, confirmed conjugation. However, because of their relatively low intensity and overlap with the CS peaks at the aliphatic region of the spectrum, the methylene protons' signals in TPP^+^ could not be differentiated.

Fig. 4(b) shows the FTIR spectra of TPP^+^-CS, TPP^+^, and CS, which range from 400 to 4000 cm^−1^. A prominent new absorption peak in the TPP^+^-CS spectra was seen at 1572 cm^−1^. This peak corresponds to the absorption bands that are linked to the C=C bonds present in the phenyl rings that are typical of the TPP^+^ moiety. Additionally, the TPP^+^-CS spectra showed extra peaks at 1642 cm^−1^ compared to the CS spectrum, suggesting the effective creation of an amide bond between the amino groups of CS and the carboxylic group of TPP^+^.

Fig. 4(c-i and ii) indicates the zeta potential of CS and CS-TPP^+^-conjugate is 34.3 mV and 44.8 mV, respectively. A significant interaction wherein the positively charged triphenyl phosphonium ion efficiently conjugates with chitosan is implied by the observed difference in zeta potentials between chitosan (CS) and its conjugate with triphenyl phosphonium ion (TPP^+^-CS).^47^

TPP method was developed and *R*^2^ was observed to be 0.999 and the regression equation was found to be 3.0109*x* + 57.32.

Once the conjugation was done, a reliable method for measuring unconjugated TPP was established using HPLC. The standard TPP^+^ chromatogram profile is shown in Fig. S2(a),† and the TPP linearity plot is given in Table S4,† which helps identify TPP concentrations that are not conjugated with line equation displayed in Fig. S2(b).† This mathematical knowledge enables precise conjugation efficiency calculations as a percentage, which is crucial for evaluating the efficacy of the conjugation process.

This study evaluated the percentage conjugation efficiency of TPP^+^ using HPLC methodology. The amount of unconjugated TPP^+^ in the reaction mixture was measured so that the percentage conjugation could be calculated by deducting it from the amount of TPP^+^ that was initially added. The resultant % conjugation for TPP^+^-CS was 45.04 ± 4% (*n* = 3, mean ± SD).

The elemental composition of TPP^+^, CS, and their conjugate (TPP^+^-CS) were analyzed using energy dispersive X-ray spectroscopy (EDAX) elemental mapping in conjunction with scanning electron microscopy (SEM) images, as shown in Fig. 4(d) The EDAX analysis verified that each structure included the expected components. In particular, the elemental analysis showed that the CS structure contains carbon, nitrogen, and oxygen (i). As predicted, TPP^+^ (ii) revealed the presence of carbon, nitrogen, oxygen, phosphorus, and bromine. The EDAX findings for the (iii) CS-TPP^+^ conjugate showed that carbon, oxygen, nitrogen, and phosphorus were present.

### 3.6 Preparation of TPP^+^-conjugated TMZ-loaded CSNPs

Conjugated nanoparticles were made utilizing the same procedure that was used to optimize the CSNPs production process by BBD. Conjugated chitosan was prepared utilizing a TPP^+^-CS conjugate polymer, then TMZ was added. Sodium tripolyphosphate (STPP) was utilized as the crosslinking agent. These conjugated nanoparticles were made using the same ionic gelation procedure that is utilized to prepare CSNPs.

### 3.7 Characterization of CSNPs and conjugated TPP^+^-conjugated TMZ-loaded CSNPs

Prepared TMZ-loaded CSNPs and TPP^+^-conjugated TMZ-loaded CSNPs were evaluated for particle size, polydispersity index, zeta potential, % entrapment, and % drug loading.

#### 3.7.1. Particle size, PDI, and zeta potential

**Fig. 5:**
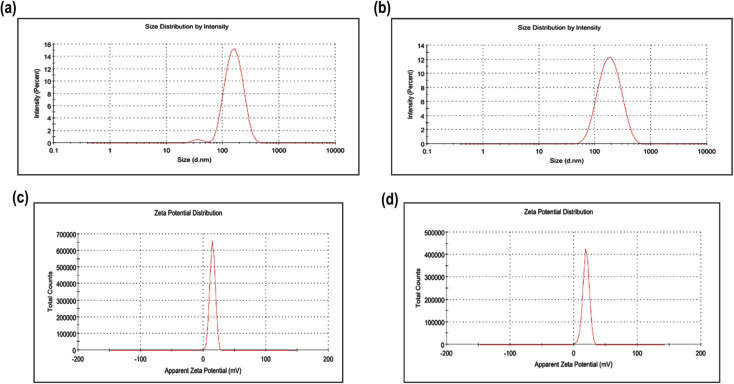
(a) Particle size plot of TMZ-loaded CSNPs, (b) particle size plot of TPP^+^-conjugated TMZ-loaded CSNPs, (c) zeta potential plot of TMZ-loaded CSNPs, (d) zeta potential plot of TPP^+^-conjugated TMZ-loaded CSNPs (all measurements were taken in triplicate with nano ZS).

The optimized formulation of TMZ-loaded CSNPs encapsulating TMZ showed a particle size of 137.5 ± 3.45 nm and a polydispersity index (PDI) of 0.245 ± 0.06 (Fig. 5(a)). On the other hand, the TPP^+^-conjugated TMZ-loaded CSNPs showed a particle size of 161.1 ± 2.56 nm and 0.145 ± 0.05 (Fig. 5(b)). The non-conjugated chitosan nanoparticles had a zeta potential of +14.6 ± 1 mV (Fig. 5(c)), while the conjugated one had a zeta potential of +18.8 ± 1 mV (Fig. 5(d)) (*n* = 3, mean ± S. D.). The optimized formulation's positively charged surface increases its propensity for interacting with negatively charged tumor cells. Furthermore, it is anticipated that the conjugated nanoparticles would target and interact with cancer cells more successfully than the non-conjugated chitosan nanoparticles due to their larger positive charge.

#### 3.7.2. Drug entrapment efficiency (% EE) and % drug loading (% DL)

An indirect technique was used to evaluate % EE of the optimized formulation of TMZ-loaded CSNPs and TPP^+^-conjugated TMZ-loaded CSNPs. The CSNPs were found to have a loading capacity of 10.76% ± 1.7% and entrapment efficiency (% EE) of 93.29% ± 3%. The percentage EE for the conjugated CSNPs was determined to be 90.07% ± 4.5%, with a loading capacity of 11.18% ± 1.4% (*n* = 3, mean ± S. D.)

### 3.8 *In vitro* drug release study

**Fig. 6:**
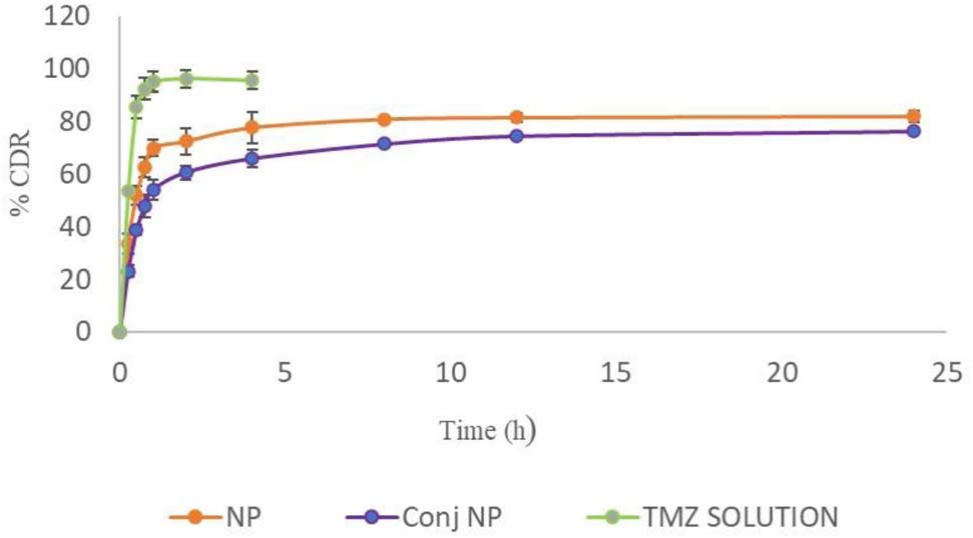
*In vitro* drug release profile of TMZ from NPs, conjugated NPs, and TMZ solution in pH 5 PBS at 200 rpm at 37 ± 1 °C (*n* = 3).

TMZ solution showed almost 95% drug release (pH 5 PBS) in 1 h while TMZ-loaded CSNPs and TPP^+^-conjugated TMZ-loaded CSNPs showed 70% and 54% drug release after 1 h, respectively, and elicited sustained release up to 24 h. Both the NPs formulations were tested for drug release kinetics at different time intervals in pH 5 PBS. Different kinetic models such as first order, zero order, Higuchi, Korsmeyer–Peppas, Hixson–Crowell, and Weibull were used to analyze the drug release data for the optimized TMZ-loaded CSNPs and conjugated CSNPs. Both the formulations' release profiles (Fig. 6), most closely followed the Weibull model, as demonstrated by the largest correlation coefficients (*R*^2^) in Tables 8 and 9. The release of a medication from a pharmaceutical dosage form is described mathematically by the Weibull release kinetics model. In situations when conventional models, such as zero-order or first-order kinetics, would not match well, this model is very helpful in characterizing drug release patterns. By allowing for parameter flexibility, the Weibull model can describe intricate release behaviors.^48^ Due to their capacity to control drug release through diffusion, degradation, and swelling processes, chitosan nanoparticles display Weibull release kinetics. This implies that the drug is delivered in two stages; first, rapid release (by diffusion), and then gradual and controlled release (probably *via* matrix disintegration).^49^ For deciding the best-fit model, 2 parameters, *viz*; AIC and MSC were taken into consideration. Akaike Information Criterion, or AIC, is a metric for assessing the relative merits of statistical models for a particular collection of data. It offers a method for choosing models by weighing the model's complexity and goodness of fit. Since a lower AIC value suggests a balance between goodness-of-fit and model complexity, it denotes a better-fitting model. A statistic called the Model Selection Criterion (MSC) evaluates a model's goodness-of-fit relative to other models. It is beneficial in pharmacokinetics and other areas where choosing the best model is essential. A greater MSC score denotes a better model-to-data fit.^50,51^

**Table 8:** Drug release kinetics of TMZ-loaded CSNPs

	Weibull	Korsmeyer–Peppas	Higuchi	Hixson–Crowell	First order	Zero order
*R* ^2^	0.9833	0.8991	−1.0499	−1.3792	0.6554	−3.103
AIC	63.4273	82.9821	117.1162	118.9038	95.7197	125.4434
MSC	2.6949	1.0653	−1.7792	−1.9281	0.0039	−2.4731

**Table 9:** Drug release kinetics of TPP^+^-conjugated TMZ-loaded CSNPs

	Weibull	Korsemeyer–Peppas	Higuchi	Hixson–Crowell	First order	Zero order
*R* ^2^	0.9915	0.928	0.0948	−0.1905	0.4242	−2.1551
AIC	44.3555	63.7604	87.0809	89.8211	82.5571	42.0267
MSC	3.5188	1.5783	−0.7537	−1.0278	−0.3014	−1.6370

### 3.9 Morphological assessment of NPs

**Fig. 7:**
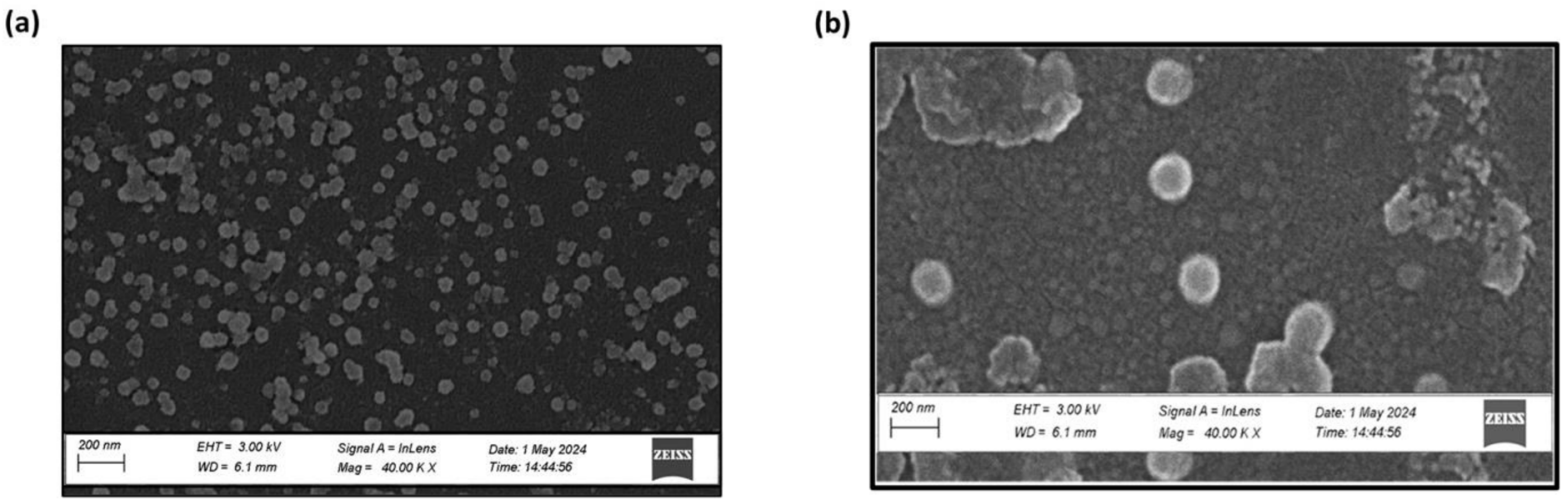
Surface morphology of (a) TMZ-loaded CSNPs, (b) TPP^+^-conjugated TMZ-loaded CSNPs showing uniform and spherical morphology.

Scanning electron microscopy (SEM) image of the optimized TMZ-loaded CSNPs & TPP^+^-conjugated TMZ-loaded CSNPs is shown in Fig. 7(a and b). The figure depicted that the particles have remarkably homogenous core structures and are spherical in shape. These nanoparticles' measured sizes, which fall between 100 and 200 nm, agree with the *Z*-average readings found using Zetasizer analysis.^52^

### 3.10 Stability study

**Fig. 8:**
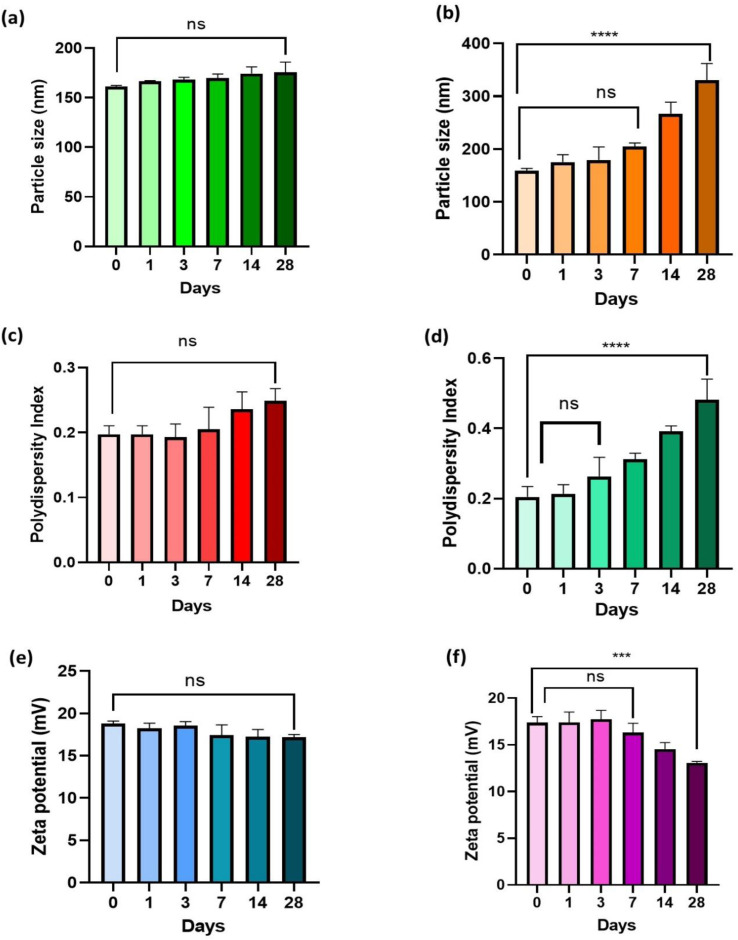
Stability data of TPP^+^-conjugated TMZ-loaded CSNPs in terms of (a) particle size when stored at 4 ± 1 °C; no statistically significant difference (ns; *p* = 0.0612) between day 0 and day 28, (b) particle size when stored at 25 ± 1 °C; no statistically significant difference (ns; *p* = 0.1257) between day 0 and day 7 while statistically significant difference (****p < 0.0001) between day 0 and day 28, (c) PDI when stored at 4 ± 1 °C; no statistically significant difference (ns; *p* = 0.1156) between day 0 and day 28, (d) PDI when stored at 25 ± 1 °C; no statistically significant difference (ns; *p* = 0.4450) between day 0 and day 3 while statistically significant difference (****p < 0.0001) between day 0 and day 28, (e) zeta potential when stored at 4 ± 1 °C; no statistically significant difference (ns; *p* = 0.1346) between day 0 and day 28, (f) zeta potential when stored at 25 ± 1 °C; no statistically significant difference (ns; *p* = 0.6284) between day 0 and day 7 while statistically significant difference (***p < 0.001; *p* = 0.0004) between day 0 and day 28 (data represented as mean ± S. D., *n* = 3, ***p < 0.001, ****p < 0.0001, ns – no statistical significant difference).

As seen in Fig. 8, the results of the stability study were evaluated in terms of zeta potential (mV), polydispersity index (PDI), and particle size (nm). The findings demonstrated that TPP^+^-conjugated TMZ-loaded CSNPs showed no significant difference at *p* > 0.05 in terms of particle size, PDI, and zeta potential at 4 ± 1 °C even after 28 days. However, at 25 ± 1 °C, particle size and PDI of TMZ-loaded CSNPs tend to increase over a period of time and a statistically significant difference was observed (*p* < 0.0001) from 0 to 28 days (Fig. 8(a–d)) while zeta potential decreased over 28 days and a significant difference was observed (*p* < 0.001) from day 0 to day 28 (Fig. 8(e and f)). The same trend was observed in the case of TMZ-loaded CSNPs stored at 25 ± 1 °C and found to be stable at 4 ± 1 °C for 28 days (Fig. S3†) (*n* = 3, mean ± SD).

### 3.11 *Ex vivo* study

**Fig. 9:**
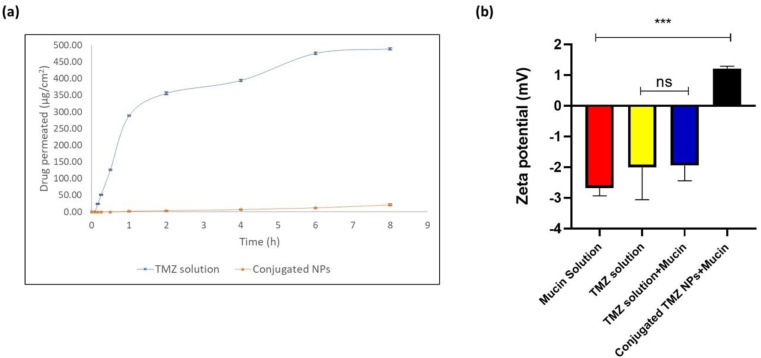
(a) *Ex vivo* drug permeation profile on goat nasal mucosa demonstrating high permeation for TMZ solution and greater retention for conjugated NPs owing to its mucoadhesion property, (b) comparative statistical analysis of zeta potential for mucoadhesion study; no statistically significant difference (ns; *p* = 0.9991) between TMZ solution and TMZ solution with mucin while statistically significant difference (***p < 0.001; *p* = 0.0002) between mucin solution and conjugated TMZ NPs with mucin (data represented as mean ± S. D., *n* = 3, ***p < 0.001, ns-no statistical significant difference).

Permeation profile was obtained for TMZ solution and optimized TPP^+^-conjugated TMZ-loaded CSNPs on an isolated nasal mucosa. The high permeation of the drug shown by TMZ solution indicated the burst release of the surface drug. The cumulative amount of TMZ permeated from TMZ solution and optimized TPP^+^-conjugated TMZ-loaded CSNPs were 383.7 ± 3.5 μg and 47.54 ± 5.2 μg, respectively, after 8 h. The TMZ solution showed enhanced drug permeation of 488.84 ± 3.64 μg cm^−2^ while conjugated NPs showed 21.04 ± 2.33 μg cm^−2^ after 8 h. Consequently, the steady-state flux for TMZ solution at 8 h was also found to be almost 30 times more when compared to conjugated CSNPs (61.10 ± 0.56 μg cm^−2^ h^−1^ for TMZ solution *vs.* 2.62 ± 0.29 μg cm^−2^ h^−1^ for conjugated CSNPs through the nasal mucosa). The results are shown in Fig. 9(a). Chitosan nanoparticles have a high mucoadhesive property, which can cause them to adhere to the nasal mucosa, potentially slowing their permeation. Nanoparticles may be surface-modified to enhance their mucoadhesive properties and interaction with the olfactory mucosa. This modification can prolong their residence time in the nasal cavity, increasing the chances of interaction with olfactory neurons.^53^

### 3.12 Mucoadhesion study

The comparative zeta potential analysis of mucin solution, TMZ solution, TMZ solution + mucin, and conjugated TMZ NPs + mucin is shown in Fig. 9(b), and is suggested to reflect the interaction between mucin, TMZ solution, and conjugated NPs.

The zeta potential of mucin solution, TMZ solution, TMZ solution + mucin, and conjugated TMZ NPs + mucin were found to be −2.67 ± 0.210 mV, −2.005 ± 0.858 mV, −1.94 ± 0.409 mV, and +1.21 ± 0.066 mV, respectively. The negative zeta potential of temozolomide in aqueous solutions is primarily due to its chemical structure, which includes ionizable functional groups like an amide group (–CONH), and a methyl group (–CH_3_) that can carry negative charges under physiological conditions. These charges affect the electric potential at the surface of the molecule or its aggregates in solution, resulting in a negative zeta potential. Further mucin solution was also found to have negative zeta potential due to sialic acid ionization. From Fig. 9(b), it was seen that there was no significant difference between TMZ solution and TMZ solution mixed with mucin implying no interaction between mucin and drug solution. On the other hand, positive zeta potential observed in the case of conjugated TMZ NPs might be due to ammonium ions (NH^3+^) present in chitosan coupled with positively charged mitochondriotropic moiety, *i.e.*, TPP^+^ which contribute towards overall positive zeta potential.^54^ There was a statistically significant difference observed between mucin solution and conjugated TMZ NPs at ***p < 0.001 implying that there might be a possibility that linked conjugated chitosan nanoparticles and mucin will have a significant electrostatic interaction decreasing the overall zeta potential of mucin.^55^

### 3.13 Cytotoxicity study

IC_50_ value, a half maximal inhibitory concentration that kills 50% of the cell, can be used to measure the potency, safety, and efficacy of the drug, though the IC_50_ value is not the sole parameter to decide the potency and safety of the compound. TMZ solution, TMZ-loaded CSNPs, and TPP^+^-conjugated TMZ-loaded CSNPs were subjected to Alamar Blue assay for cytotoxicity. Data was processed in GraphPad Prism 8.0.2, and the IC_50_ value was determined using non-linear regression analysis. It was observed that the IC_50_ value of TMZ solution was found to be 1646 ppm after 24 h of treatment. For TMZ-loaded CSNPs formulation, the IC_50_ value was observed at 17.06 ppm while for TPP^+^-conjugated TMZ-loaded CSNPs, it was 16.05 ppm after 24 h of treatment. The overall results indicated that conjugated NPs were more cytotoxic (around 100 times) than plain TMZ solution signifying their potency in killing GB cells.

### 3.14 ROS study

**Fig. 10:**
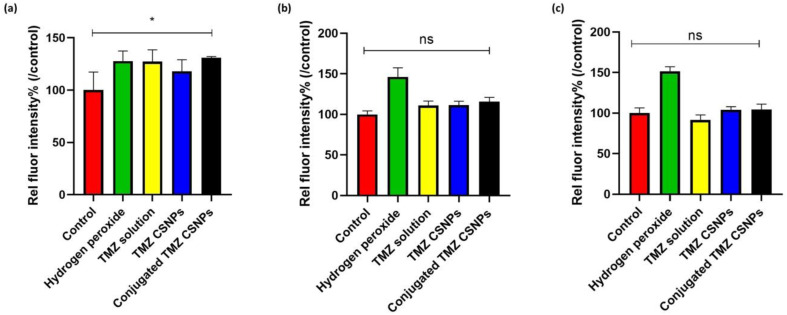
Comparative statistical analysis of relative fluorescence intensity normalized to control (%) (a) post 3 h treatment; statistically significant difference (*p < 0.05; *p* = 0.0467) between control and conjugated TMZ CSNPs, (b) post 6 h treatment; no statistical difference (ns; *p* = 0.0973) between control and conjugated TMZ CSNPs, (c) post 12 h treatment; no statistical difference (ns; *p* = 0.9038) between control and conjugated TMZ CSNPs (data represented as mean ± S. D., *n* = 3, *p < 0.05, ns – no statistical significant difference).

Intracellular redox homeostasis significantly influences the regulation of signaling cascades that govern apoptosis and proliferation in malignant cells. Several established chemotherapeutic agents, including TMZ, possess the capacity to induce ROS, thereby causing DNA damage.^56^ Our findings also demonstrated that there was a significant increase in intracellular ROS production in TPP^+^-conjugated TMZ-loaded CSNPs compared to a control group, after 3 h of treatment (Fig. 10(a)). However, over a period, the extent of the increase in fluorescence intensity, which is directly proportional to ROS production, decreased. (Fig. 10(b) and (c)). It is important to understand that both normal and cancer cells rely on Nrf2 transcription network to counteract oxidative stress. Nrf2 regulates a suit of antioxidant genes, ensuring redox balance. Due to its protective function, Nrf2 activity is upregulated in GB cells after TMZ exposure.^57^ TMZ is also known to modulate cell viability by influencing autophagy, and hypoxia-inducible factor-1α activity. Various reports suggested that chaperone-mediated autophagy (CMA) is responsible for effective TMZ treatment through the promotion of apoptosis. CMA activity is initiated by a transient surge of ROS within cytoplasm, originating from mitochondria subsequent to endoplasmic reticulum stress.^58^ This might be the possible reason behind the transient increase of ROS levels up to 3 h.

### 3.15 Cellular uptake study

Qualitative drug internalization into glioblastoma cell lines, *i.e.*, U87MG cells, was performed with rhodamine B-labeled formulation. The experiment was done after 2 h and 4 h of treatment with formulation and it was seen that fluorescence intensity of rhodamine B was increased from 2 h to 4 h of treatment (Fig. S4†). It was also observed that compared to the TMZ solution, TPP-conjugated TMZ-loaded CSNPs showed higher rhodamine B intensity in both instances, *viz.*, 2 h and 4 h, indicating greater cellular uptake of conjugated NPs. A similar finding was reported by Mohammadian *et al.*^46^

## 4. Conclusion

Mitochondria play a crucial role in cell apoptosis, and their dysfunction is associated with numerous diseases, including cancer. This has led to the development of mitochondria-targeted drug delivery systems. While most early efforts focused on attaching targeting moieties to therapeutic or sensing molecules, nanoparticle-based systems have gained significant attention in recent years. This study reports the development and optimization of chitosan nanoparticles with temozolomide chosen as a chemotherapeutic drug. A QbD strategy was utilized with Taguchi design for screening variables followed by BBD design for optimization purposes, leading to improved entrapment efficiency, decreased particle size, and PDI. We then successfully synthesized mitochondria-targeted chitosan-TPP^+^ conjugated nanoparticles with carbodiimide reaction and validated them with varied techniques like FTIR, EDAX, NMR, HPLC, and zeta potential measurements. The *in vitro* release profile of conjugated nanoparticles in acidic pH was comparable to that of unconjugated nanoparticles suggesting a negligible impact of conjugation procedure. Stability study indicated that conjugated nanoparticles were stable at 4 °C for 28 days. *Ex vivo* study on goat nasal mucosa proved that conjugated CSNPs exhibited increased retention on mucosa, thereby reducing the drug permeation by virtue of stronger electrostatic interaction between negatively charged mucin and positively charged TPP and CS in conjugated nanoparticles. This finding was also supported by a mucin interaction study and evidenced by the alteration of zeta potential. *In vitro* cell line studies showed greater cytotoxicity potential for conjugated nanoparticles than free TMZ solution on U87MG cells. Further conjugated nanoparticles showed increased intracellular ROS generation compared to control cells, thereby proving to cause damage to malignant cells. In conclusion, this study successfully developed and optimized mitochondria-targeted CSNPs for temozolomide delivery, demonstrating enhanced nasal mucosa retention and improved physicochemical properties, stability, and cytotoxicity against glioblastoma cells offering a promising approach for glioblastoma therapy.

## Data availability

The data supporting this article have been included as part of the ESI.†

## Author contributions

TGA and Aakanchha J. conceptualized and designed this study. AD and TGA were involved in all the experimental studies and manuscript writing/editing, leading to the completion of the manuscript. TGA performed analyzed and interpreted the results. Ankit J. reviewed and edited the manuscript. Aakanchha J. reviewed the manuscript and supervised the study.

## Conflicts of interest

The authors declare no conflict of interest.

## Supplementary Material

RA-014-D4RA04748F-s001
