# Supplementary material for: Quality-by-design-engineered mitochondrial targeted nanoparticles for glioblastoma therapy
Source: RSC Adv. 2024 Oct 28;14(46):34100–18. doi: 10.1039/d4ra04748f (PMC11513785; doi:10.1039/d4ra04748f)
Supplement: RA-014-D4RA04748F-s001 [file RA-014-D4RA04748F-s001.pdf]

Supplementary Information (SI) for RSC Advances.
This journal is © The Royal Society of Chemistry 2024

Quality-by-Design-engineered mitochondrial targeted nanoparticles for glioblastoma therapy

Akanksha Dahifale[a#], Tejas Girish Agnihotri[a#], Ankit Jain*[b], Aakanchha Jain*[a]

[a]Department of Pharmaceutics, National Institute of Pharmaceutical Education and Research (NIPER)-Ahmedabad, Palaj, Gandhinagar-382355, Gujarat, India

[b]Department of Pharmacy, Birla Institute of Technology & Science, Pilani-333031, Rajasthan, India

# These authors contributed equally

***Corresponding authors**

Dr. Aakanchha Jain (jainaakanchha83@gmail.com; aakanchha.jain@niperahm.res.in)

Dr. Ankit Jain (ankit.j@pilani.bits-pilani@ac.in)

Supplementary Figures:

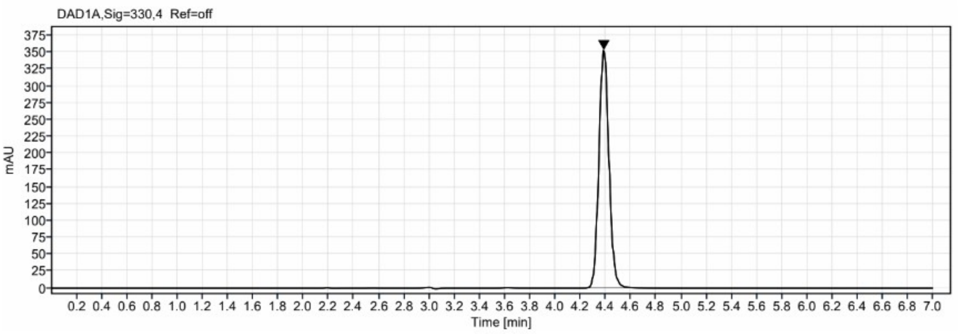

Fig. S1(a) HPLC chromatogram of Temozolomide (TMZ) with detection wavelength at 330 nm and retention time of 4.3 min and run time of 7 min

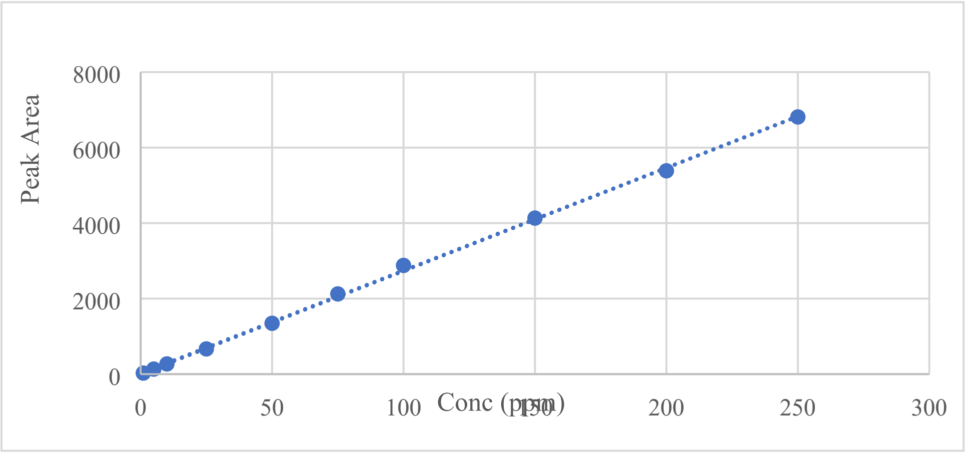

Fig. S1(b) Calibration curve and regression equation of TMZ in Milli-Q water with linearity ranging from 1 µg/mL to 250 µg/mL and R^2 value of 0.9993

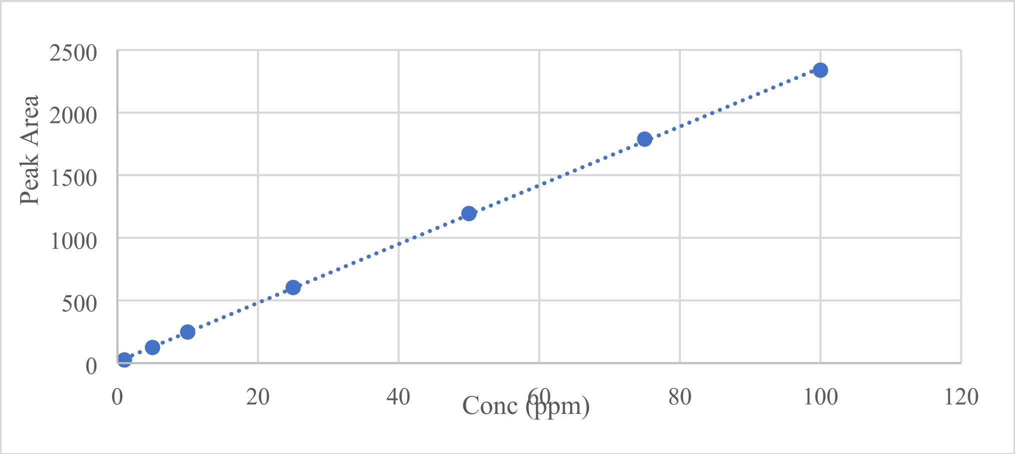

Fig. S1(c) Calibration curve and regression equation of TMZ in Phosphate Buffer Saline (PBS; pH 5) with linearity ranging from 1 µg/mL to 100 µg/mL and R^2 value of 0.9998

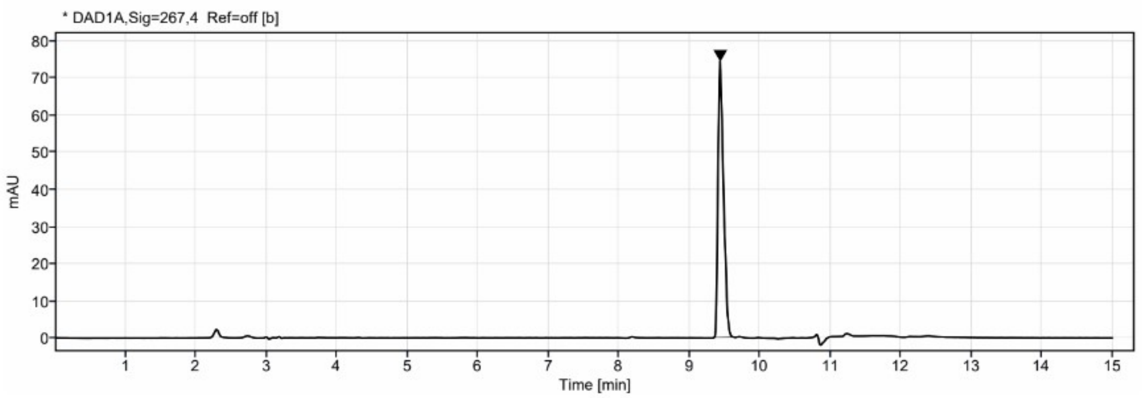

Fig. S2(a) HPLC chromatogram of TPP with detection wavelength at 267 nm and retention time of 9.4 min and run time of 15 min

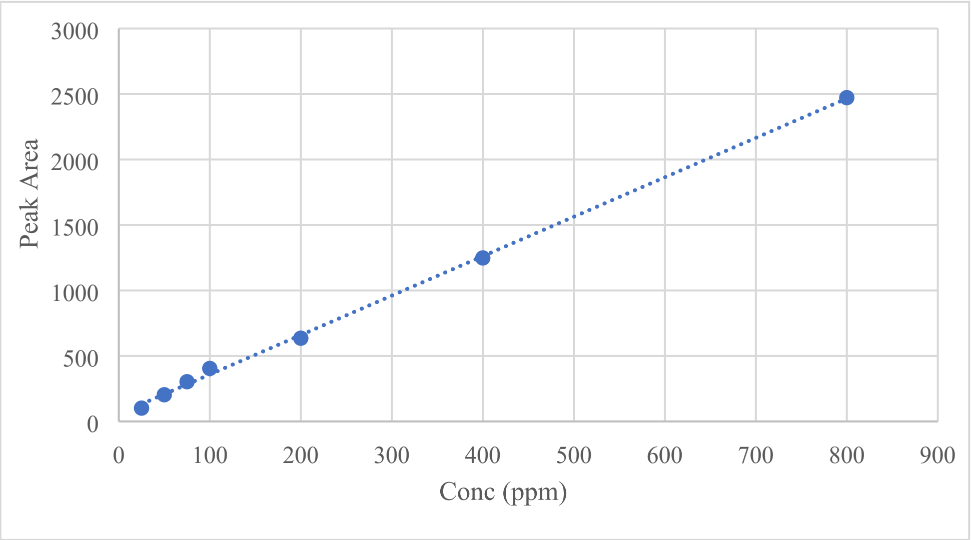

Fig. S2(b) Calibration curve and regression equation of TPP with linearity ranging from 25 ppm to 800 ppm and R^2 value of 0.999

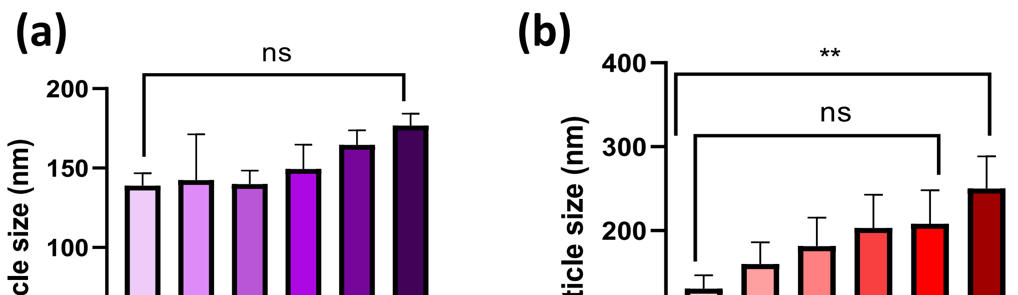

Fig. S3 Stability data of TMZ-loaded CSNPs (a) Particle size stored at 4±1˚C; no statistically significant difference (ns) between day 0 and day 28 at $p=0.0786$ (b) Particle size stored at 25±1˚C; statistically significant difference (**$p<0.01$; $p=0.0091$) between day 0 and day 28 (c) PDI stored at 4±1˚C; no statistically significant difference (ns) between day 0 and day 28 at $p=0.0672$ (d) PDI stored at 25±1˚C; statistically significant difference (**$p<0.01$; $p=0.0033$) between day 0 and day 28 (e) Zeta potential stored at 4±1˚C; no statistically significant difference (ns) between day 0 and day 28 at $p=0.4274$ (f) Zeta potential stored at 25±1˚C; statistically significant difference (*$p<0.05$; $p=0.0101$) between day 0 and day 28 (Data

represented as mean ± S.D., n=3, multigroup analysis was made by Ordinary one Way-ANOVA (Parametric test) followed by Tukey's post hoc test; *p<0.05, **p<0.01, ns-no statistical significant difference)

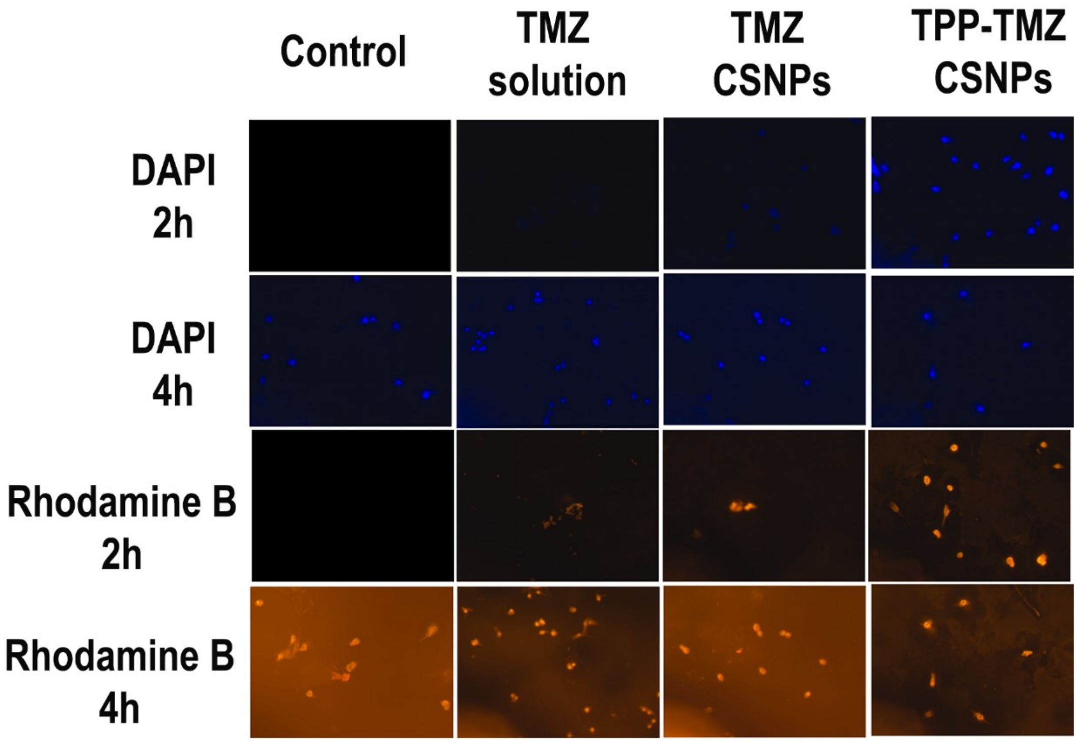

Fig. S4: Cellular uptake images with rhodamine B staining and DAPI as counterstain (Nuclei staining) at 2h and 4h of formulation treatment, viz., control (without treatment, TMZ solution, TMZ-loaded CSNPs and TPP-conjugated TMZ-loaded CSNPs) confirming greater cellular uptake by U87 cells for conjugated NPs than TMZ solution

Supplementary Tables

QTPP	Target	Justification
Administration Route	Intranasal	It allows the fast drug passage directly to the brain, avoiding the need ~~of crossing~~to cross the BBB.
Clinical use	Glioblastoma treatment	Temozolomide is the first-line agent for treating GBM, by alkylation of adenine/guanine residues, leading to DNA damage through futile repair cycles and eventual cell death of cancerous cells.
Drug Delivery system	TPP^{+}-Conjugated Nanoparticles	Conjugated nanoparticles selectively target the mitochondria of cancerous cells by differentiating the mitochondrial potential of cancerous cells and have sizes < 200 nm, and polydispersity index of less than 0.25 (homogeneity) promoting the direct passage to the brain through the trigeminal and olfactory nerves.
Pharmaceutical Dosage Form	Aqueous Dispersion	Facilitates nasal application through a spray device.

Table S1: Quality target profile (QTPP) of TPP^{+}-conjugated TMZ-loaded CSNPs

Factor	DOF	Sum of squares	F ratio	P value
TMZ Conc	1	4.945E+06	19.60	0.0068
Stirring speed-2	1	3.017E+06	11.96	0.0181
Residual	5	1.261E+06	-	-
Cor total	7	9.223E+06	-	-

Table S2~~1~~(a) ANOVA analysis of particle size in Taguchi design

Table S2~~1~~(b) ANOVA analysis of PDI in Taguchi design

Source	Sum of Squares	df	Mean Square	F-value	p-value	
Model	7.34	3	2.45	16.25	0.0105	significant
A-Chitosan conc	2.71	1	2.71	17.99	0.0133	
F-Stirring speed-2	1.60	1	1.60	10.60	0.0312	
G-Time-2	3.03	1	3.03	20.15	0.0109	
Residual	0.6020	4	0.1505			
Cor Total	7.94	7				

Table S2~~1~~(c) ANOVA analysis of % entrapment efficiency in Taguchi design

**Source**	Sum of Squares	df	Mean Square	F-value	p-value	
Model	0.2713	1	0.2713	8.52	0.0267	significant
B-TMZ conc	0.2713	1	0.2713	8.52	0.0267	
Residual	0.1911	6	0.0318			
Cor Total	0.4624	7				

Table S32(a) ANOVA Table of particle size in BBD

Source	Sum of Squares	df	Mean Square	F-value	p-value	
Model	59658.75	4	14914.69	3.01	0.0401	significant
A-Chitosan conc	21204.67	1	21204.67	4.28	0.0505	
B-TMZ Conc	1845.39	1	1845.39	0.3727	0.5478	
C-Stirring Speed-2	2278.28	1	2278.28	0.4601	0.5047	
D-Time-2	37139.20	1	37139.20	7.50	0.0120	
Residual	1.089E+05	22	4951.94			
Lack of Fit	59210.05	19	3116.32	0.1880	0.9922	not significant
Pure Error	49732.67	3	16577.56			

Cor Total	1.686E+05	26				

Table S3~~2~~(b) ANOVA Table of PDI in BBD

Source	Sum of Squares	df	Mean Square	F-value	p-value	
Model	0.2632	8	0.0329	6.35	0.0006	significant
A-Chitosan conc	0.0113	1	0.0113	2.19	0.1564	
B-TMZ Conc	0.0017	1	0.0017	0.3209	0.5780	
C-Stirring Speed-2	0.0644	1	0.0644	12.43	0.0024	
D-Time-2	0.0009	1	0.0009	0.1829	0.6740	
AB	0.0682	1	0.0682	13.17	0.0019	
AC	0.0227	1	0.0227	4.38	0.0508	
C^2	0.1182	1	0.1182	22.81	0.0002	
D^2	0.0228	1	0.0228	4.39	0.0505	
Residual	0.0932	18	0.0052			
Lack of Fit	0.0385	15	0.0026	0.1404	0.9966	not significant
Pure Error	0.0548	3	0.0183			
Cor Total	0.3565	26				

Table S32(c) ANOVA Table of % entrapment efficiency in BBD

Source	Sum of Squares	df	Mean Square	F-value	p-value	
Model	1483.15	5	296.63	5.43	0.0023	significant
A-Chitosan conc	33.63	1	33.63	0.6157	0.4414	
B-TMZ Conc	1.30	1	1.30	0.0237	0.8790	
D-Time-2	141.71	1	141.71	2.59	0.1222	
BD	423.75	1	423.75	7.76	0.0111	
A^2	882.31	1	882.31	16.15	0.0006	
Residual	1146.96	21	54.62			
Lack of Fit	1094.74	18	60.82	3.49	0.1653	not significant
Pure Error	52.23	3	17.41			
Cor Total	2630.12	26				

Table S4: Linearity range and respective peak area of TPP solution

Sr. No.	Concentration (ppm)	Peak Area
1	25	101.639
2	50	203.772
3	75	303.238
4	100	404.502
5	200	635.424
6	400	1248.345
7	800	2472.301
